# A methodological guideline for consciousness assessment via neural electrophysiological activity

**DOI:** 10.1186/s40779-025-00682-4

**Published:** 2025-12-12

**Authors:** An-An Ping, Long-Zhou Guan, Yong Wang, Sheng Yang, Chao Yang, Xiao-Qing Hu, Yi-Heng Tu, He Chen, Wei-Guang Li, Xiao-Li Li

**Affiliations:** 1 State Key Laboratory of Cognitive Neuroscience and Learning, Beijing Normal University, Beijing, 100875 China; 2 Institute of Advanced Technology, South China University of Technology, Guangzhou, 511442 China; 3 Department of Rehabilitation Medicine, Zhujiang Hospital, Southern Medical University, Guangzhou, 510280 China; 4 Department of Psychology, the State Key Laboratory of Brain and Cognitive Sciences, the University of Hong Kong, Hong Kong SAR, 999077 China; 5 HKU‑Shenzhen Institute of Research and Innovation, Shenzhen, 518057 Guangdong China; 6 State Key Laboratory of Cognitive Science and Mental Health, Institute of Psychology, Chinese Academy of Sciences, Beijing, 100101 China; 7 Department of Psychology, University of Chinese Academy of Sciences, Beijing, 101408 China; 8 School of Automation Science and Engineering, South China University of Technology, Guangzhou, 510641 China; 9 State Key Laboratory for Quality Ensurance and Sustainable Use of Dao-Di Herbs, Artemisinin Research Center, and Institute of Chinese Materia Medica, China Academy of Chinese Medical Sciences, Beijing, 100700 China; 10 Pazhou Lab, Guangzhou, 510335 China

**Keywords:** Consciousness, Electroencephalogram, Temporo-spatio-spectral analysis, Sleep, General anesthesia, Disorders of consciousness

## Abstract

**Background:**

Physiological, pharmacological, and pathological alterations of consciousness provide critical windows into its neural substrates. Given the inherent complexity and multidimensionality of consciousness, defining quantitative, dynamic signatures of neural activity, and translating them into clinically applicable tools remains challenge. This study aimed to build an electroencephalography (EEG)-based methodological guideline for clinical consciousness assessment.

**Methods:**

EEG signals were systematically categorized across periodic and aperiodic activity, connectivity and network topology, spatiotemporal dynamics, self-organized criticality, and transcranial magnetic stimulation (TMS)-evoked responses. These biomarkers were mapped onto a conceptual framework of consciousness, comprising wakefulness and internal/external awareness, based on their validation across clinical conditions. The discriminative efficacy of various biomarkers was then evaluated across 4 independent datasets.

**Results:**

Integrated EEG features each captured distinct yet complementary dimensions of consciousness, supporting a unified neurophysiological architecture underlying diverse alterations of consciousness. Spectral power and peak frequency tracked the loss of consciousness during propofol anesthesia and sleep. Steeper aperiodic slopes, loss of frontoparietal connectivity, disrupted small-world organization, and reduced effective dimensionality were particularly effective in distinguishing minimally conscious state (MCS) from unresponsive wakefulness syndrome (UWS). Additionally, spatiotemporal patterns exhibited consciousness-specific alterations, with both pharmacological and pathological alterations influencing specific microstate dynamics.

**Conclusions:**

Synthesizing integrated neural dynamics and multidimensional consciousness, this guideline establishes both methodological and theoretical foundations for translating neurophysiological biomarkers into clinical applications. While this work advances both conceptual clarity and practical methodology, large-scale validation across expanded clinical cohorts, experimental models, and multimodal platforms is essential to fully establish causal linkages and translational utility.

**Supplementary Information:**

The online version contains supplementary material available at 10.1186/s40779-025-00682-4.

## Background

Consciousness is a richly multidimensional construct that encompasses subjective experience, self-awareness, attention, memory, and emotion, emerging from complex interactions across multiple cognitive systems. Over the past decades, advances in neuroimaging have accelerated research on the neural correlates of consciousness (NCC), the minimal neural mechanisms sufficient to support specific conscious experiences [1–4]. First, contemporary neuroscience investigates NCC along 2 primary dimensions [1, 2, 5–7]. The content of consciousness (awareness), which reflects the specific information accessible to subjective experience at any given moment, can itself be subdivided into internal awareness (e.g., emotions, mind-wandering) and external awareness (e.g., connection to the environment allowing perception of external stimuli) [7, 8]. Second, the level or state of consciousness (wakefulness), spans a continuum from full alertness to drowsiness and even unconsciousness [5], providing the foundational scaffold upon which conscious content is structured [6]. Although classic paradigms such as binocular rivalry and visual masking can identify neural differences between awareness and unawareness through introspective reports, a major challenge remains in assessing consciousness when introspection is unavailable, as occurs in many clinical conditions.

In clinical contexts, consciousness can be broadly modulated by physiological, pharmacological, and pathological factors. Physiological alterations refer to natural variations in normal bodily functions, characterized by spontaneous, reversible, and rhythmic fluctuations in consciousness [9]. During the sleep-wake cycle, progression into non-rapid eye movement (NREM) stages is characterized by a gradual decline in responsiveness and sensory disconnection, reflecting the attenuation of wakefulness and awareness that culminates in deep NREM stage 3 (N3). In contrast, during rapid eye movement (REM) sleep, despite the low level of wakefulness and suppressed external sensory input, there is heightened cholinergic activity and cortical activation resembling the awake state [10]. This distinctive neural configuration supports internally generated conscious experiences, most notably vivid dreaming [11]. Although dreaming is not exclusive to REM sleep, we focus here on the contrast between typically dreamless deep NREM sleep and dream-associated REM sleep for simplicity.

Pharmacological alterations involve the controlled modulation of consciousness using anesthetic agents in clinical settings [12, 13]. General anesthesia (GA) produces a reversible state of unconsciousness characterized by preserved physiological homeostasis, along with immobility, amnesia, and unresponsiveness [14]. The neural correlates of GA-induced unresponsiveness vary with the anesthetic mechanism. For instance, propofol enhances the inhibitory effect on postsynaptic γ-aminobutyric acid type A (GABA_A_) receptors, reducing neuronal excitability and typically resulting in complete loss of consciousness (LOC) with low wakefulness and awareness [14]. In contrast, N-methyl-D-aspartic acid receptor antagonists such as ketamine induce a dissociative anesthetic state (referred to here as disconnected consciousness) [15], marked by reduced wakefulness and profoundly impaired behavioral interaction with the external environment, yet often accompanied by dream-like experiences resembling REM sleep [8, 13, 16].

Pathological disorders of consciousness (DOC) typically result from traumatic brain injury, stroke, or hypoxic-ischemic events. Patients with DOC are clinically classified according to 3 core syndromes: coma, characterized by the absence of spontaneous eye opening, sleep-wake cycles, and any behavioral responsiveness, refer to complete loss of wakefulness and awareness [17, 18]; unresponsive wakefulness syndrome (UWS), marked by restored wakefulness behaviorally defined by eye opening and sleep-wake cycles, but no detectable awareness [19]; and the minimally conscious state (MCS), presenting intermittent but reproducible signs of self or environmental awareness [20]. In approximately 15–25% of behaviorally unresponsive DOC patients, with a condition termed cognitive motor dissociation, brain activation in response to motor commands can be detected using functional magnetic resonance imaging or electroencephalography (EEG) [17]. The high prevalence of REM sleep in MCS (approximately 88%) suggests the preservation of internal awareness [21], though this remains difficult to assess directly. Since these latent forms of awareness often cannot be expressed through voluntary movement, they pose a significant risk of misdiagnosis [22].

**Fig. 1:**
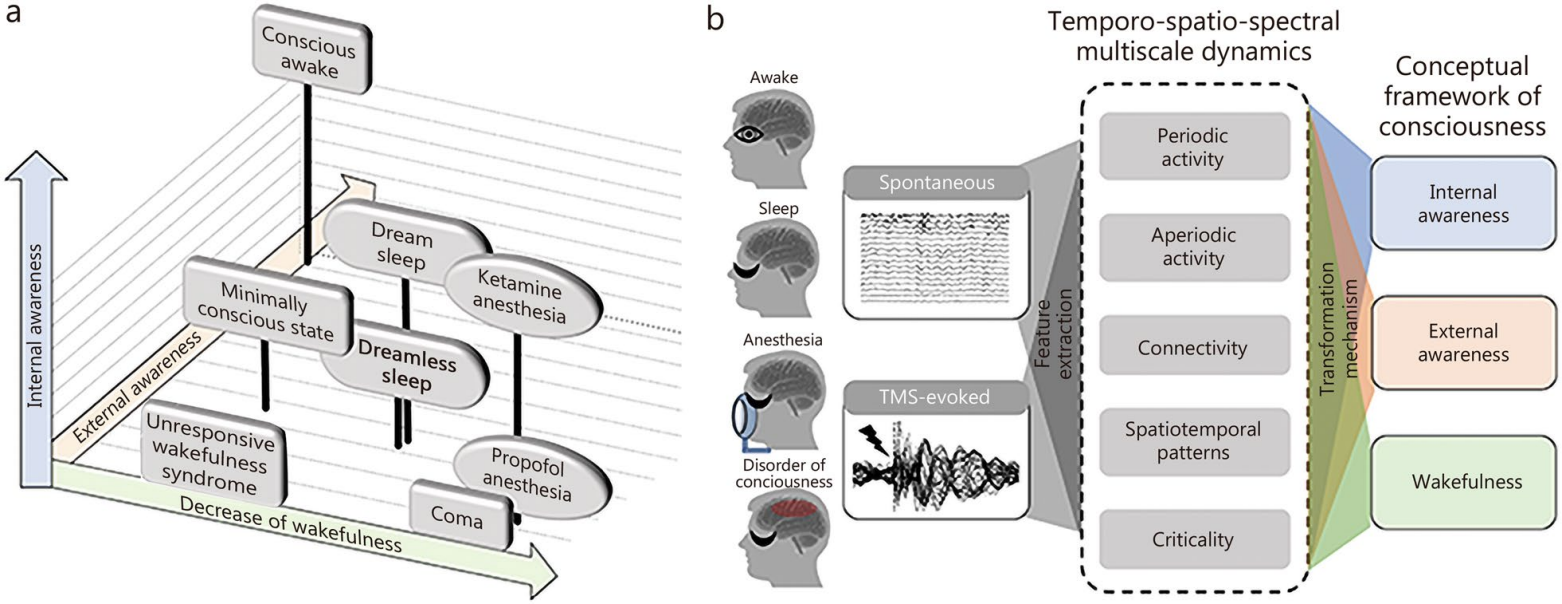
Integration of neural electrical dynamics into the conceptual framework of consciousness. **a** The conceptual frameworks of consciousness comprise the 3 dimensions: “wakefulness”, “internal awareness”, and “external awareness”. In a normal conscious awake state, all 3 dimensions operate at their upper limit. Unresponsive wakefulness syndrome retains high wakefulness without internal or external awareness, whereas minimally conscious state demonstrates some capacity for internal and external awareness. Dream-associated rapid eye movement (REM) sleep and ketamine-induced anesthesia with dream reports display internal awareness alongside attenuated external awareness and wakefulness. Dreamless deep non-rapid eye movement (NREM) sleep, propofol-induced anesthesia, and coma present all 3 dimensions at their minimum level. **b** Spontaneous or transcranial magnetic stimulation (TMS)-evoked neuroelectrical activity provides a basis for the objective characterization and assessment of consciousness

Critically, observations from these clinical conditions reveal that unresponsiveness does not necessarily imply LOC [16, 23]. To better characterize such states, we employ an integrative three-dimensional framework of consciousness comprising “wakefulness”, “internal awareness” and “external awareness” [8]. This framework helps to distinguish between connected consciousness (high levels across all dimensions) and states of unresponsiveness, including 1) disconnected consciousness (low wakefulness/external awareness but preserved internal awareness) and 2) complete LOC (low levels across all dimensions). Figure 1 illustrates the physiologically, pharmacologically, and pathologically altered states of consciousness based on the three-dimensional framework.

Although behavioral scales such as the Coma Recovery Scale-Revised (CRS-R) remain the current clinical standard for consciousness assessment [24], they lack the sensitivity to detect covert awareness in behaviorally unresponsive patients, such as those with cognitive motor dissociation [25]. Neurophysiological recordings, ranging from single-neuron activity and local field potentials to scalp EEG, offer more direct and temporally precise probes. Several theoretical frameworks offer predictions about the neural dynamics underlying fluctuations in consciousness [26]. Integrated information theory posits that consciousness emerges from complex and irreducible neural causal interactions centered on the posterior cortical areas [27, 28]. Global neuronal workspace theory (GNWT) proposes that consciousness arises when information is globally ignited and broadcast across a distributed cortical workspace [5, 29]. The temporo-spatial theory of consciousness emphasizes that nested spatiotemporal patterns shape the conscious experience [30]. These distinct theories of consciousness, converge on the need for quantitative, dynamic, and multiscale neurophysiological markers of consciousness, yet challenges remain in translating them into clinically applicable tools.

In principle, ideal unified EEG-based approaches for consciousness assessment should generalize across physiological, pharmacological, and pathological conditions. In practice, however, individual EEG metrics often diverge across different clinical conditions. These inconsistencies stem from methodological heterogeneity and the multidimensional nature of consciousness, where different dimensions may map onto distinct electrophysiological signatures. It is therefore essential to identify the mapping relationships between electrophysiological signatures and the different dimensions of consciousness, and to develop a multi-dimensional electrophysiological signature framework for consciousness assessment.

To bridge this gap between theory and clinical application, we propose a practical methodological guideline for EEG-based consciousness assessment in clinical settings. First, we categorize EEG features along temporal, spatial, and spectral dimensions. These signatures are then integrated into a three-dimensional framework of consciousness grounded on their well-validated associations with various conscious states, such as those observed during NREM/propofol-induced LOC, REM/ketamine induced disconnected consciousness, and DOC in MCS/UWS patients (Fig. 1 and Table 1). By bringing the multiscale neural dynamics and the multidimensional organization of consciousness to the foreground, this unified interpretation framework helps reconcile contradictions in the existing literature and elucidate common principles underlying physiologically, pharmacologically, and pathologically altered consciousness. It provides researchers and clinicians with a practical tool to select appropriate electrophysiological biomarkers based on specific clinical needs and to integrate multi-dimensional indicators to assess consciousness.

**Table 1:** Consciousness alterations in sleep, anesthesia, and in patients with disorders of consciousness

States/Dimensions	Wakefulness	External awareness	Internal awareness
Physiological alterations			
Awake state	Present	Present	Present
Deep NREM sleep	Absent	Possible	Absent
REM sleep	Absent	Possible	Present
Pharmacological alterations			
Propofol anesthesia	Absent	Absent	Absent
Ketamine anesthesia	Absent	Absent	Present
Pathological alterations			
Unresponsive Wakefulness syndrome	Present	Absent	Absent
Minimally conscious state	Present	Possible	Possible

*NREM* non-rapid eye movement, *REM* rapid eye movement

## EEG-based approaches for consciousness assessment

Building upon the multidimensional framework of consciousness and its alterations in clinical conditions introduced previously, we first systematically categorize and detail electrophysiological features and then explore their mapping to specific dimensions of consciousness based on their verified assessment utility.

**Fig. 2:**
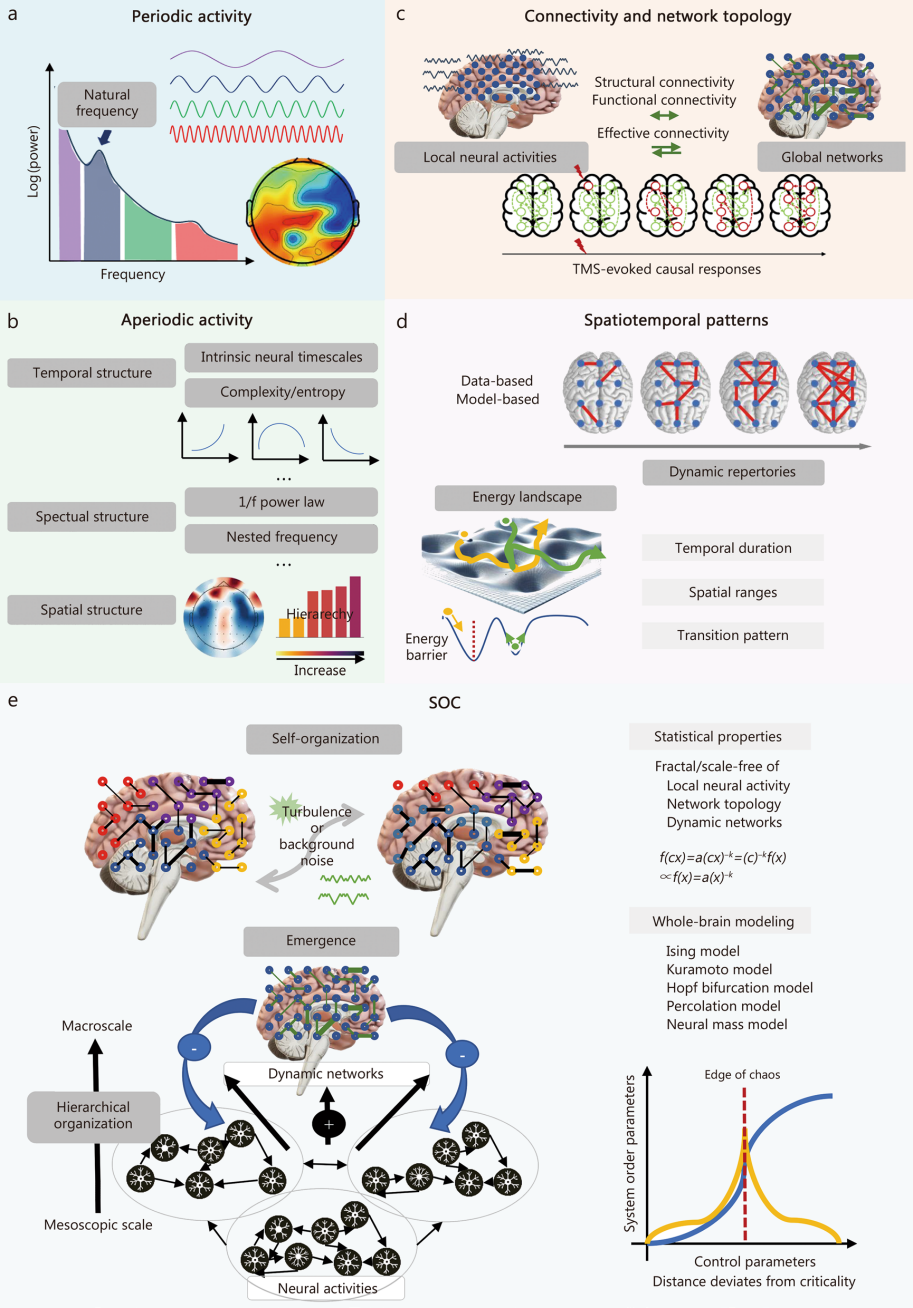
Multiscale electrophysiological signatures for consciousness assessment. Periodic activity (**a**) and aperiodic activity (**b**) reflecting fundamental electrophysiological components at local brain regions. Periodic activity, such as alpha rhythms and natural frequency, reflects canonical neural oscillations, while aperiodic activity captures broadband background dynamics, typically characterized by the 1/f component and intrinsic timescales. **c** Connectivity and network topology. The interplay of periodic or aperiodic activities across local regions reflects long-range communication, generating functional and effective (causal) neural circuits. These circuits form structured topological networks on a whole-brain scale. **d** Spatiotemporal patterns. Beyond static networks, spatiotemporal patterns such as metastable dynamics and transient network configurations capture the fluid balance between integration and segregation of neural activity over time. Spatiotemporal patterns, such as metastable states and energy landscapes, capture the time-varying characteristics of large-scale network dynamics. **e** Self-organized criticality (SOC). Under the framework of complex systems science, the principle of SOC provides a unifying lens to interpret how these temporal, spatial, and spectral features interact across scales, balancing periodic and aperiodic activity, network integration and differentiation, as well as stability and flexibility of information processing. TMS transcranial magnetic stimulation

Our analysis focuses on 6 complementary EEG perspectives to characterize neural activity across multiple scales, ranging from local electrical activity and neural circuits to large-scale networks and their dynamic evolution [30]. These perspectives include: periodic activity, aperiodic activity, connectivity and network topology, spatiotemporal patterns, self-organized criticality, and transcranial magnetic stimulation (TMS)-evoked causal responses (Fig. 2).

### Periodic activity associated with consciousness

Periodic activities arise from synchronized neuronal electrical activity and can be decomposed into distinct frequency bands through spectral analysis techniques, including the Fourier transform, wavelet transform, and empirical mode decomposition [31] (Fig. 2a). These oscillations are characterized by 2 fundamental parameters: 1) amplitude, reflecting the strength of synchronized activity at a given frequency, and 2) phase, indicating the temporal progression within an oscillatory cycle [31].

Delta oscillations (1−4 Hz) originate primarily from thalamic activity and are normally suppressed by cholinergic and noradrenergic arousal systems. High-amplitude delta oscillations (HADOs), ranging from tens to hundreds of microvolts, emerge prominently during deep NREM sleep, propofol-induced LOC [32, 33], and in DOC patients (particularly in UWS patients) [34]. These oscillations typically occur globally and are most pronounced in frontal regions. Transient HADOs have been shown to serve as a marker for LOC following bolus propofol administration, which can result in a rapid transition into unconsciousness [35]. In contrast, under slow-infusion protocols, loss of behavioral responsiveness is more closely associated with increased beta power rather than HADOs [36]. As the effect-site concentration (Ce) of propofol rises further, slow-wave activity (SWA, 0.5–1.5 Hz, delta occasionally including frequencies < 1.0 Hz [37]) becomes dominant, particularly in frontal regions, marking the transition into loss of awareness and thalamocortical isolation [36]. This SWA occurs asynchronously across cortical regions, reflecting massive synchronization at local regions but disrupted long-range network integration [38]. Therefore, anesthesia-induced changes in delta oscillations are influenced by multiple factors, including the rate of drug administration (slow vs. rapid infusion), the types of delta activity (e.g., narrowband SWA vs. broader 1–4 Hz), and differences between states (disconnected consciousness vs. LOC). In addition, study combining EEG with dream reports found that increased delta power (0.5–4.5 Hz) in the posterior cortical “hot zone” (parieto-occipital regions) serves as a robust marker for the absence of dream experience [39]. Therefore, findings from both anesthesia and sleep support the notion that increased delta oscillations are associated with LOC, especially loss of internal awareness. However, some studies have also reported increased delta oscillations in states of disconnected consciousness [40, 41] and awake states with cognitive disorders [42]. For instance, there appear to be at least 2 distinctive clusters of delta waves during REM: 2.5–3.0 Hz oscillations localized in frontal-central regions and < 2.0 Hz oscillations localized in medial-occipital regions [43]. This prompts us to examine the possibility that certain types of delta oscillations (regional, frequency-specific) may not indicate LOC, but rather signs of disconnection from the external environment such as functional decoupling between thalamic and sensory cortical regions [37].

Alpha oscillations (8–13 Hz) are the most dominant neural rhythm in the awake states and may originate from thalamocortical interactions, modulating perception through neural mechanisms such as active inhibition and attentional sampling. In DOC patients, decreased alpha-beta power and increased delta power correlate with the severity of consciousness impairment [44]. Absolute alpha power even exhibited the highest diagnostic performance for distinguishing MCS from UWS patients [45]. However, another study suggests that alpha power may only be suppressed in ischemic-hypoxic UWS patients, failing to discriminate cases with other etiologies [46]. The high diagnostic performance of alpha power in some studies may arise from the high prevalence of hypoxic patients [44, 45], necessitating a reevaluation of its diagnostic utility across diverse etiological contexts. Furthermore, alpha activity undergoes a characteristic anteriorization during propofol-induced LOC [47–49] and NREM sleep [50], with power and coherence decreasing in occipital regions while increasing in frontal regions. This pattern likely represents a composite of 2 distinct processes, including the suppression of the occipital-dominant alpha oscillation associated with relaxed wakefulness; and the simultaneous emergence of forehead-dominant spindle activity (9–14 Hz waves occur in 1 to 2 s bursts) [51–53]. The maximization of the instantaneous alpha-band power has been reported as a robust marker of loss of behavioral responsiveness [53]. The origins of this alpha anteriorization are still debated [48, 54], and recently a human intracranial EEG study proposes that alpha anteriorization may be attributed to 2 different thalamo-cortical loops, whereby propofol disrupts the alpha coherent network between the thalamus and somatosensory-related cortex while inducing alpha coherence between the cognition-related thalamic nuclei and frontal cortex [55]. Therefore, alpha-band anteriorization is likely an epiphenomenon, a measurable component of a broader neurophysiological reorganization involving thalamocortical inhibition and functional disconnection [48, 54]. Although interventional studies indicate that neither suppressing alpha activity [56] nor enhancing spindle activity [57] alters states of unresponsiveness, current evidence remains insufficient to establish causal relationships due to methodological heterogeneity across studies [58]. The precise role of frontal spindles in consciousness regulation requires further investigation through rigorously controlled paradigms. This frontal enhancement of alpha power can only be observed during LOC induced by propofol anesthesia and NREM sleep, and is absent in states where internal awareness is preserved, such as REM sleep and ketamine-induced anesthesia [59–61]. This contrast suggests that the global reorganization of alpha activity, of which anteriorization is a part, could be a promising signature of LOC, particularly for the loss of internal awareness, even if not its direct driver.

Gamma oscillations (30–150 Hz) are theoretically linked to conscious access according to GNWT [5], a theory supported by early visual masking paradigms showing that high gamma power correlates with conscious awareness [62]. Sustained or even increased gamma power has been observed in widespread cortical and subcortical regions during both REM sleep [61] and ketamine-induced anesthesia [60, 61, 63, 64], suggesting it’s neurophysiologically associated with preserved internal awareness. Reduced gamma power (30–90 Hz) generally observed during NREM sleep [65] and propofol-induced LOC [66] further supports that reduced gamma power is related with LOC, specifically the loss of internal awareness. However, a paradoxical increasing in gamma power (25–40 Hz) has also been reported under propofol-induced LOC [32]. These conflicting findings have often been attributed to differences in anesthesia depth, frequency band definitions, or contamination by muscular and ocular artifacts in EEG signals [67]. It has also been proposed that certain types of gamma-band activity may be insufficient to sustain conscious experience [68, 69]. Sub-bands of gamma oscillations generated by distinct mechanisms exhibit differential sensitivity to consciousness [66, 70, 71]. Low-gamma activity may be enhanced under anesthesia due to GABA_A_ receptor-mediated hyperpolarization of fast-spiking interneurons, resulting in localized cortical hyperactivity [72]. The visual stimulation experiments also indicated that, compared to high-frequency oscillations, low-gamma oscillations do not necessarily correspond to conscious perception [69]. High-gamma activity (> 40 Hz), which is more closely linked to functions such as motor control, working memory, and sensory perception [73], tends to be suppressed through broader network inhibition [65]. Moreover, gamma responses exhibit pronounced regional heterogeneity [32, 39, 74]. Increased gamma activity in posterior cortices combined with decreased delta activity commonly predicts the presence of internal awareness in sleep [39]. In contrast, intrahemispheric gamma coherence may even be higher in LOC during NREM sleep than in waking [74], and increased gamma activity is often observed during propofol anesthesia in frontal regions such as the anterior cingulate and prefrontal cortices, localized in components of the default mode network [32], implying that even elevated gamma in these areas may not suffice to maintain consciousness, especially in the presence of slow waves [39]. In addition, non-verbal report paradigms indicate that gamma activity in the sensory cortex primarily reflects local stimulus processing that increases the probability of conscious access, positioning it as a prerequisite for rather than a direct correlate of conscious experience [75]. This emphasizes the importance of designing new experimental paradigms that distinguish conscious perception from related pre-conscious (such as attention selection) [2] and post-conscious (such as decision-making) processes [76], thus serving to verify the role that gamma oscillations play in awareness emergence. Therefore, although gamma oscillations are generally regarded as highly relevant to external/internal awareness, their consciousness-specific effects are best interpreted within an integrated framework that incorporates frequency subbands, regional specificity, and recording techniques.

Natural frequencies are the preferred oscillatory frequencies of cortical regions and form spatial gradients following the cortical hierarchy [77, 78]. The individualized natural frequency can be quantified through several approaches: 1) individualized dominant frequency, computed as the peak frequency or spectral centroid within a specific frequency band [e.g., alpha peak frequency (APF)]; 2) instantaneous frequency, such as frequency sliding computed as the first derivative of the phase angle time series [79]; or 3) TMS-evoked oscillations [77]. These natural frequencies show task-dependent modulation [79], enabling adaptation to the structural sensory inputs and the temporal patterns of intrinsic information transfer. In the awake state, the homogeneity of APF across regions potentially facilitates global synchronization, in contrast, anesthesia-induced LOC features frontal predominance and elevated inter-regional variability of APF [80]. This shift from a homogeneous to heterogeneous APF distribution may be related to the disruption of large-scale neural synchrony [80]. Moreover, APF measured with frequency sliding is significantly slower in both ketamine- and sevoflurane-induced unresponsiveness, reflecting the diminished capacity for temporal input processing related to external connection.

### Aperiodic activity associated with consciousness

Aperiodic activity is another fundamental component of brain electrophysiology, characterized by irregular, non-repetitive temporal waveforms and broadband spectral distribution without dominant frequency peaks (Fig. 2b). This activity supports dynamic and flexible conscious information processing, while exhibiting spatially organized gradients across cortical regions that mirror the functional architecture of the brain [78].

#### Temporal structures in EEG associated with consciousness

To process continuous external inputs with nested temporal scales, such as the hierarchical structure of speech spanning phonemes, words, and sentences, neural activity is itself organized into a hierarchy of intrinsic neural timescales (INTs) [81, 82]. These timescales shape information processing through mechanisms of temporal integration and segregation [83].

Quantitative characterization of INTs has been achieved using autocorrelation window (ACW) analysis and detrended fluctuation analysis (DFA), which capture long-range temporal correlations in neural signals [84] (Fig. 2b). ACW measures the time windows required for a signal’s autocorrelation function to decay below a defined threshold (typically 0.5), reflecting the temporal persistence of neural activity; DFA evaluates self-similarity organization in nonstationary signals by measuring the power-law relationship between fluctuation magnitude and observation timescales, where a DFA index > 0.5 indicates the presence of long-range correlations. In general, longer INTs reflect more sustained neural activity, which is often associated with enhanced information integration, whereas shorter INTs favor the segmentation and rapid processing of transient stimuli [85]. A hierarchical organization of INTs has been consistently observed in both human and non-human primates, with shorter INTs in unimodal sensorimotor regions (periphery) and progressively longer INTs in higher-order transmodal areas (core, e.g., anterior cortex and temporoparietal junction) [81, 83, 86]. These hierarchical gradients facilitate the simultaneous segregation and integration of information across functionally specialized regions [78, 86]. Disruption to this INT’s hierarchy is evident in states of unresponsiveness. Ketamine- and sevoflurane-induced anesthesia [84, 87, 88], NREM and REM sleep [84, 89], and DOC patients present prolonged INTs compared to the awake state. In addition, the peripheral-to-core gradient of INTs [90] and the correlation between ACW and APF are disrupted during anesthesia and DOC [88]. These findings suggest that the proper organization of INTs may serve as a necessary condition for wakefulness or external information processing, and its disruption underlies various states of unresponsiveness, including LOC and disconnected consciousness.

Information-theoretic complexity metrics provide complementary insights by quantifying the unpredictability or randomness of neural signals [91, 92]. These metrics can be categorized by their analytical scope. Time-domain entropy measures (relevant dimensions, approximate entropy, permutation entropy, fuzzy entropy, symbolic entropy, multiscale entropy, Lempel-Ziv complexity, etc.) and temporal-frequency entropy measures (response entropy, state entropy, wavelet entropy, Hilbert spectrum entropy) capture the temporal complexity of neural activity, and have shown satisfactory performance in monitoring the depth of anesthesia [91]. Spatiotemporal complexity metrics, including amplitude joint entropy and synchrony joint entropy [93], extend the above entropy analyses to multi-channel recordings by reconstituting the multi-channel EEG data into a unified representation, thereby assessing complexity across both time and space. These metrics primarily reflect the randomness of a signal, and are known as type I complexity [94] (Fig. 2b). Type I complexity typically declines from the awake state to deep NREM sleep before rebounding during REM sleep, mirroring the trajectory of conscious experience [95, 96], and can effectively differentiate NREM sleep from REM sleep and awake state [97]. Measurements such as algorithmic complexity and permutation entropy of neural signals, especially over posterior regions, also effectively predict behavior [98] and the prognosis of DOC patients, and can even identify potential consciousness in UWS patients [97, 99]. However, type I complexity decreases under propofol-induced anesthesia [91, 93, 100–102] but remains unchanged or even increases during ketamine-induced anesthesia [103] and REM sleep [96]. This divergence suggests that a reduction in type I complexity (randomness) does not indicate disconnected consciousness but rather indicates the absence of internal awareness.

The maximum randomness of signals does not imply information capacity. For instance, Gaussian white noise exhibits maximum randomness but carries no usable information [104]. To address this limitation, type II complexity was proposed. By multiplying disorder and a distance function of the current state of the system from complete disorder, its metrics quantify information capacity which peaks around the point where the system is in a state that is neither excessively regular nor purely random [104, 105] (Fig. 2b). Type II complexity exhibits consistent reduction during unresponsiveness in REM sleep [106] and both anesthetic states [105], indicating that it likely reflects a wakefulness-specific effect. Therefore, the presence of internal awareness may preserve neural randomness [23, 107], but the potential lack of structural or functional organization limits meaningful information storage and transmission. These phenomena could be interpreted by “the edge of chaos” hypothesis [106], which posits that consciousness depends on a critical phase transition between neural flexibility and the capacity for accessing meaningful information [108–110], whereas alterations of consciousness tend toward either rigidly order (e.g., NREM, generalized seizures) or unpredictable randomness (e.g., REM, anesthesia) [106].

#### Spectral structures in EEG associated with consciousness

Long-range temporal correlations of neural activity, which exhibit a distinctive scale-free distribution in the spectral domain with a power-law gradient from slow to faster frequencies, are widely observed across spatial scales, from single-unit spiking to macroscopic EEG/magnetoencephalography recordings [111]. This power-law distribution can be quantified by 1/f exponents of power spectral density (PSD). Steeper 1/f exponents indicate more low-frequency activities, theorized according to the Wiener-Khinchin theorem to reflect greater cortical inhibition [112] or to predict shorter ACW. Many advanced approaches have been developed to parameterize the 1/f exponent, including coarse graining spectral analysis (CGSA), irregular resampling auto-spectral analysis (IRASA), and fitting oscillations and one-over-F (FOOOF) [113]. Region- and frequency-dependent alterations in 1/f patterns occur in states of unresponsiveness [114]. During NREM sleep [84, 96] and under propofol-induced LOC [87, 115], the 1/f exponents (1–40 Hz) exhibit a global increase relative to the awake state, with pronounced elevation in prefrontal regions. A similar increase is observed in UWS patients when contrasted with those in MCS [46]. Conversely, ketamine-induced anesthesia with dreaming reports demonstrates parieto-occipital domination of 1/f modulations but reveals a critical frequency-dependent dissociation, with increased exponents at low frequencies (1–20 Hz) [84, 116] and decreased exponents at higher frequencies (20–40 Hz) [116]. Another frequency-specific phenomenon occurs during wakefulness-to-REM transitions, where high-frequency (30–45 Hz) 1/f exponents exhibit the strongest augmentation at REM [114] while low-frequency (1–30 Hz) exponents peak at NREM [84, 96]. Taken together, these findings indicate that 1/f exponents not only index unresponsiveness but also differentiate disconnected consciousness from complete LOC. Their frequency- and region-specific modulation patterns thus help delineate different dimensions of consciousness [114].

Beyond the scale-free nature of the whole spectrum [111], cross-frequency coupling analyses reveal how different frequencies couple or nest with each other [117]. The most studied form, phase-amplitude coupling (PAC), examines how the phase of slower oscillations modulates the amplitude of faster oscillations. PAC demonstrates particularly state-dependent reorganization, wherein anterior-to-posterior delta-alpha PAC strengthens during deep NREM sleep [118], while posterior delta-gamma [119] and cortical-to-hippocampal theta-gamma PAC dominate in the awake state and during REM sleep [120, 121]. Preserved PAC patterns may underlie memory consolidation during REM sleep [120, 121]. The slow wave-alpha PAC also serves as a valuable indicator for tracking propofol-induced LOC and recovery of consciousness (ROC) [33]. During transitions into and out of unconsciousness, alpha amplitude maximizes at the phase troughs of low-frequency oscillations, predominantly in the anterior cingulate and frontal regions, while during sustained LOC, alpha amplitude is greatest at the low-frequency phase peaks, occurring across widespread cortical areas. However, the “slow oscillation-alpha” PAC pattern shows limited sensitivity for detecting accidentally connected consciousness during ketamine-induced anesthesia [122]. The specificity of PAC to distinct dimensions of consciousness necessitates further investigation, integrating broader conscious state datasets and diverse parameter selections.

### Connectivity and network topology associated with consciousness

The capacity of the brain for information integration emerges from precisely coordinated interactions among distributed neural populations (Fig. 2c). Functional connectivity (FC) captures statistical dependencies between neural signals through various measurements. Phase- and amplitude-based synchronization metrics comprise phase lag index (PLI), phase locking value (PLV), amplitude envelope correlation (AEC), and coherence. Measures based on information theory, such as mutual information, weighted symbolic mutual information (wSMI), and permutation cross-mutual information (PCMI), provide complementary insights into information sharing across large-scale brain networks [123, 124]. Effective connectivity represents the causal influence between neural populations [124]. Time-domain approaches like Granger causality, directed transfer function, symbolic transfer entropy (STE), and permutation conditional mutual information identify predictive relationships, whereas frequency-domain techniques, including partial directed coherence, phase slope index, and directed phase lag index (dPLI), reveal frequency-specific information flow. Model-based methods such as dynamic causal modeling (DCM) incorporate biophysical constraints to infer network architecture.

Based on the functional/effective connectivity capturing neural coordination patterns, network topology describes system-level organization principles operating within the 2 competing demands of functional segregation for specialized local processing, and functional integration of parallel modules for global information synthesis [125]. Functional integration refers to the ability to rapidly exchange and coordinate specialized information from distributed modular regions. It is typically quantified as both the ease of information transfer, using metrics like characteristic path length [126] and global efficiency [127], and as the complexity of large-scale network coordination, using metrics like matrix decomposition and principal component analysis [128]. Functional segregation supports specialized local processing through clustered connectivity patterns, measured via clustering coefficient [126], local efficiency [127], and modularity [129]. Moreover, small-world architecture, calculated as the ratio of average clustering coefficient to characteristic path length, represents an efficient balance between local specialization and global integration, enabling the maximization of information communication at minimal wiring cost [126]. Scale-free organization, evidenced by a power-law distribution of degree, reflects the fractal hierarchical structure of network connections [130, 131]. Generally, higher fractal dimensionality correlates with greater complexity, where small perturbations can induce substantial macroscopic changes. The coexistence of these properties allows neural networks to maintain robust yet adaptable functional architecture.

During transitions from awake states into unconsciousness (e.g., NREM sleep [132] or propofol-induced LOC) and from MCS to UWS [98, 123], key electrophysiological changes include: 1) shift of predominant alpha coupling (measured by PLI) from temporal-parietal to prefrontal regions [50, 133]; 2) attenuation of anterior-posterior theta/alpha connectivity (quantified via PLI and wSMI) [98]; and 3) increased prefrontal delta synchronization (assessed by PLI) [98, 132]. Notably, prefrontal delta-band PLV is also enhanced during both dexmedetomidine (another inhibitory anesthetic) and ketamine anesthesia, concurrent with increased intra-hemispheric phase offsets and decreased inter-hemispheric ones [134], suggesting this may be a common feature across various unresponsiveness. Moreover, comparative analyses suggest that amplitude-based synchrony metrics (e.g., AEC) outperform phase-based metrics (e.g., weighted PLI) [135] in distinguishing LOC from awake states or disconnected consciousness [136], and UWS from MCS [136], particularly in the delta-theta band, rather than slower oscillations (1.0–2.5 Hz) or in the alpha band. Amplitude-based gamma couplings show hypersynchrony patterns during propofol-induced LOC and NREM sleep [32, 136, 137], and maintain intermediate levels resembling the awake state during REM sleep and ketamine anesthesia [137]. However, gamma PCMI disrupts in the frontal, parietal, and occipital regions during unresponsiveness, regardless of anesthetic agent [107], highlighting the impact of methodological choices. These findings indicate that frequency- and measure-dependent connectivity patterns serve distinct functional roles. Certain patterns, such as gamma and prefrontal alpha hypersynchrony, are associated with a loss of internal awareness, whereas other patterns, such as delta phase locking, track declines in wakefulness or predict the breakdown of sensory processing.

Effective connectivity captures consistent patterns of altered information flow across various states of unresponsiveness. STE, dPLI, and DCM reveal reduced frontal-posterior feedback connectivity, while preserving feedforward and thalamo-cortical connectivity during propofol- and ketamine-induced anesthesia [60, 138–141] and in DOC patients (especially UWS patients) [142]. Granger causality analyses also show global loss of cortical information flow during propofol-induced LOC, especially frontal-posterior [143]. The asymmetric structure of frontoparietal forward (bottom-up) and backward (top-down) connectivity may be a wakefulness-specific feature or may relate to external connectedness [140].

The transition to LOC is reliably associated with fundamental reorganization of brain network topology [144], with particularly prominent disruptions in parietal networks [107, 144, 145]. In the awake state, the brain networks typically maintain optimal small-world properties [80, 146]. Propofol-induced LOC significantly reduces alpha network asymmetry, driving shifts of the central hub from parietal to frontal regions [80, 144]. This reorganization of network topology involves enhanced local processing (increased clustering coefficients, elevated modularity, and prolonged characteristic path length [107, 147, 148]), alongside impaired global integration (decreased global efficiency [146] and effective dimensionality (ED) [128]). Such topological alterations have also been partially reported across multiple states of altered consciousness, including ketamine anesthesia [107], deep NREM sleep [128, 149–151], and REM sleep [152], and demonstrate discriminative power in distinguishing MCS from UWS [153–156], then typically revert to baseline levels following ROC [148]. Together, these findings suggest that frequency-specific network impairments, particularly in the alpha band, appear to track unresponsiveness, potentially reflecting underlying changes in wakefulness and external connectedness. Furthermore, although REM sleep [152] and ketamine anesthesia [107] significantly increase clustering coefficients, they maintain an unchanged characteristic path length in low-frequency, gamma, and broadband networks. This preservation of integrated network architecture despite predominant local connectivity suggests that sparse long-range connections may sustain frequency-specific small-world properties in disconnected consciousness.

### Spatiotemporal patterns associated with consciousness

Neural activity is hierarchically organized across different temporal, spectral, and spatial scales to adapt to and learn from the ever-changing external environment, and then to guide future actions [110, 149, 157]. In higher-order theory, this hierarchical structure likely contributes to the transformation of low-level perceptual signals into high-level elemental representations [158]. Furthermore, it facilitates the selection and propagation of local networks by global ignition according to the GNWT [5, 29] and aligns with neural integration and differentiation in the integrated information theory [27].

Large-scale neural networks continuously reconfigure connectivity patterns into transient functional states, forming a dynamic repertoire, a structured yet flexible set of metastable configurations [159] (Fig. 2d). A metastable configuration may refer either to an instantaneous, whole-brain topographic map of voltage values that remain stable for approximately 60 to 100 ms before rapidly transitioning to another configuration, such as in typical microstate analysis, or to a dynamic functional connectivity (dFC) calculated within an appropriate time window (the calculation of the FC is shown in “Connectivity and network topology associated with consciousness” section). Therefore, each configuration captures both the spatial organization and temporal persistence of neural activity [160]. These configurations are typically bounded in number and exhibit spontaneous inter-configurational transitions driven by stochastic dynamics to maintain optimal spatial organization. Computational approaches such as K-means clustering and hidden Markov models are used to characterize these recurring states and their temporal transitions [161].

The results can be represented as trajectories or manifolds in a low-dimensional state space or energy landscape (e.g., via principal component analysis or Laplacian eigenmaps) [162, 163] (Fig. 2d), and then further analyzed through metrics such as dimensionality (the minimal number of independent neural modes or degree of freedom required to represent non-redundant information in temporal evolution) [128], occupancy (the prevalence of specific states), transition probability (the likelihood of state shifts), entropy (the predictability of state changes) [164] and other statistical properties, such as the power-law distribution of dwell time [165].

The dFC of the awake state is dominated by long-distance coordination, where balanced positive and negative connections support both high modularity and global efficiency [166]. This spatiotemporal configuration fundamentally differs from states of unresponsiveness. Across unconscious conditions, including NREM sleep [167], propofol-induced LOC [164, 168], and in DOC patients [164, 166], dFC more frequently converge toward a less efficient architecture, characterized by the disappearance of long-range negative correlations, deterioration of small-world properties, and constricted dynamic repertoires. Notably, this repertoire reduction manifests as decreased temporal variability/flexibility localized in the posterior cingulate, prefrontal, and parietal cortex [164, 169]. Intriguingly, the fundamental scale-free temporal organization of dFC remains preserved during both the awake state and propofol anesthesia, despite significant modification in local connection strength and duration parameters [165]. This preserved scale-free temporal architecture may represent a critical factor enabling a reversible state of unconsciousness. Furthermore, the diffusion graph embedding algorithm distinguishes the cortical regions corresponding to different dimensions of consciousness, with UWS and propofol-induced LOC showing reduced gradients and occurrence rates in transmodal/unimodal networks, while ketamine-induced disconnected consciousness affects visual and somatomotor networks [159]. Collectively, the spatial complexity of neural network architecture, coupled with appropriate temporal variability in dynamic functional repertoire, appears critical for sustaining the continuous flow of conscious experience [159, 161].

### Self-organized criticality (SOC) associated with consciousness

From the perspective of complex systems science, the theory of SOC describes spontaneous evolution toward critical boundaries between system phases (e.g., liquid to gas) [104]. This provides a unifying framework for understanding how macroscopic hierarchical dynamics emerge from multiple spatiotemporal scales nested in a scale-free way (Fig. 2e).

The emergence of complexity observed near phase transition critical points in collectively biological behaviors [170, 171], has been considered to be a mechanism for the formation of higher-order neural systems capable of complex computation arising from local neuronal circuits with well-defined functions [172] (left panel of Fig. 2e). The neural systems may benefit from the optimal information processing capacity in these critical states [108, 160]. Specifically, emergence maximizes the diversity of dynamic repertoires [108, 109, 160], transmission efficiency [173], and functional connection diversity [110, 174, 175], without compromising the stability of transmission patterns [171]. Therefore, multiscale neural processes may operate in a (near-) critical way between order and disorder, stability and flexibility, or modularization and global integration, to process information efficiently and to adapt flexibly to environmental changes [176, 177]. Deviations from criticality impair this capacity, whereby excessively ordered states hinder adaptation to changes in the external environment, and highly random states that change continuously prevent stable long-term memory and effective information transmission.

Statistical analyses that quantify deviations from criticality based on the detection of scale-free characteristics (fractal) in the time process or network structure (right panel of Fig. 2e) include DFA [89], fractal dimension, maximum Lyapunov exponent, branching parameter, and the 0−1 chaos test [106]. At the global level, “neuronal avalanche” posits that local activity beyond threshold propagates through the system, generating large-scale “avalanches” with power-law distributed sizes and durations [177, 178]. However, the presence of power-law distributions alone does not confirm criticality [179, 180]. Complementarily, the critical states could be theoretically established through the Ising model, Kuramoto model, Hopf bifurcation model, Percolation model, or neural mass model [106, 175, 178, 181–183]: when the macroscopic order parameters (e.g., coherence, mutual information, phase-locking value, complexity) reach the optimal regime, such as at the peak of an inverted U-curve or the inflection point of a sigmoidal curve (right panel of Fig. 2e). Therefore, the divergence between empirical neural signatures and the ideal predictions of optimal equilibrium provides theoretical deviation from criticality due to pharmacological or pathological disturbances.

SOC suggests that the awake state resides on the chaotic edge of order and disorder (quantified by the 0–1 chaos test), facilitating maximal information richness, while physiological, pharmacological, or pathological perturbations shift brain dynamics away from this optimal critical regime [106, 178, 182]. The duration of neural avalanches displays similar fractal intermittency and long-range correlations (DFA) during the awake state and REM sleep, whereas NREM sleep exhibits fractal properties only at short timescales, approaching randomness at longer timescales [89, 184]. This suggests that while short-range interactions persist, global dynamics may fragment into localized, independent functional units during LOC [89, 184]. The deviation from criticality coefficient based on neural avalanches has outperformed periodic indicators in distinguishing the awake state from NREM sleep [185]. However, the fractal property of avalanche sizes remains consistent across both awake state and NREM sleep [89, 184]. Similar scale-free properties of temporal organization [165] and topological structure [131] have been observed during propofol-induced LOC. This may reflect an ongoing process of adaptive reconfiguration in neural activity during reversible LOC, potentially paving the way for the recovery of consciousness. In addition, model-based analyses indicate that both propofol- and ketamine-induced unresponsiveness deviates from criticality, manifesting a phase transition of the global network [186], reduced dynamic instability [187] and temporal variability [182, 187], altered functional-structural coupling [182, 188] and diminished information richness [106]. The fold bifurcation model captures the critical transition from wakefulness to sleep onset [181]. Deviations from criticality have also been demonstrated in DOC patients, as quantified by neural avalanches [189] and dimensionality [190], suggesting deviation from the optimal regime of network transformation [182, 191] and information richness [106].

Similar to alpha frontalization, delta/gamma power, type I complexity, and small-worldness, critical distance can distinguish between complete LOC and disconnected consciousness within unresponsiveness. Furthermore, like INTs, 1/f exponents, information richness, and effective connectivity, critical distance could further differentiate disconnected consciousness from the awake state. Collectively, these findings indicate that criticality likely represents a salient and fundamental feature of consciousness.

### TMS-evoked causal responses associated with consciousness

Assessing brain network response to external perturbations provides a robust approach for detecting NCC. By delivering controlled magnetic pulses to the cortex, TMS-EEG probes the brain’s inherent causal interactions within and between cortical regions, bypassing subcortical pathways such as thalamic gating and brainstem arousal systems [192]. This enables a direct assessment of cortical excitability and functional integration, providing a comprehensive measure of network dynamics that reflect changes in wakefulness [192] (Fig. 2c).

First, TMS-evoked potentials capture the cortical excitation-inhibition dynamics. Early components (0–50 ms post-stimulus) reveal a positive relationship with motor evoked potentials, reflecting cortical neuronal excitability; the N100 component indicates γ-aminobutyric acid type B (GABA_B_)-mediated cortical inhibition [193, 194].

Second, TMS-EEG maps the causal information flow across cortical regions [132, 195–197]. During the awake state, TMS elicits rapidly propagating cortical activation that engages distributed brain regions beyond the stimulation site, sustaining widespread recurrent activity for approximately 300 ms [196]. In contrast, NREM sleep is characterized by amplified and prolonged early TMS-evoked potential, and rapidly decaying spatial propagation limited to local cortical areas [196]. These properties can also serve as a viable marker of residual consciousness in DOC patients [197], reflecting impaired cortical effective connectivity and compromised capacity for global information integration during LOC.

Third, natural frequencies detected by TMS-EEG reveal important regional variations in intrinsic processing timescales, with alpha oscillations in the occipital cortex [78], beta oscillations in the parietal cortex, and high beta and gamma oscillations in the frontal cortex [77]. This gradient facilitates efficient information transfer [77] but collapses into uniform, high-amplitude slow oscillations (< 1 Hz) across cortical regions during NREM sleep [196] and anesthesia-induced LOC [80]. Comparable frequency reductions (up to 10 Hz) also occur in subcortical and frontal regions in stroke [198–200] and schizophrenia [201]. Similar to INTs hierarchy, gradients of natural frequencies facilitate global neural synchronization, potentially representing a fundamental mechanism sustaining wakefulness and supporting the processing of external information.

Finally, the spatiotemporal complexity analysis of TMS-induced activity can characterize dynamic patterns of integration and differentiation across varying states of consciousness. The perturbational complexity index (PCI) and its subsequent refinement are typical measures of spatiotemporal complexity, respectively quantifying complexity by concatenating multidimensional data into a single-dimensional vector or by employing principal-component recursive analysis [97, 102]. Reduced PCI values effectively track LOC during NREM sleep [97], propofol anesthesia [99, 102], and in DOC patients [97, 99]. However, PCI remains elevated during ketamine-induced anesthesia and REM sleep [102], and is closely associated with dream reports, suggesting preserved neural variability linked to internal awareness.

## Clinical validations

To demonstrate the practical utility of the proposed guideline for consciousness assessment, clinical validations were conducted using 4 empirical EEG datasets encompassing anesthesia, sleep, DOC, and TMS-EEG (Additional file 1: Materials and methods).

### Periodic activity across propofol-induced anesthesia and sleep stages

Periodic activity was analyzed using dataset 1 to investigate propofol-induced alterations in consciousness, including awake, mild sedation, moderate sedation, and recovery states. Parallel analysis was performed on dataset 2, which captured physiological changes in consciousness across wakefulness, NREM2, NREM3, and REM sleep. PSD was computed using the Fourier transform with a sliding window of 10 s without overlap. The relative power and center frequency were extracted across delta (1−4 Hz), alpha (8−13 Hz), and low gamma (30−45 Hz) bands.

**Fig. 3:**
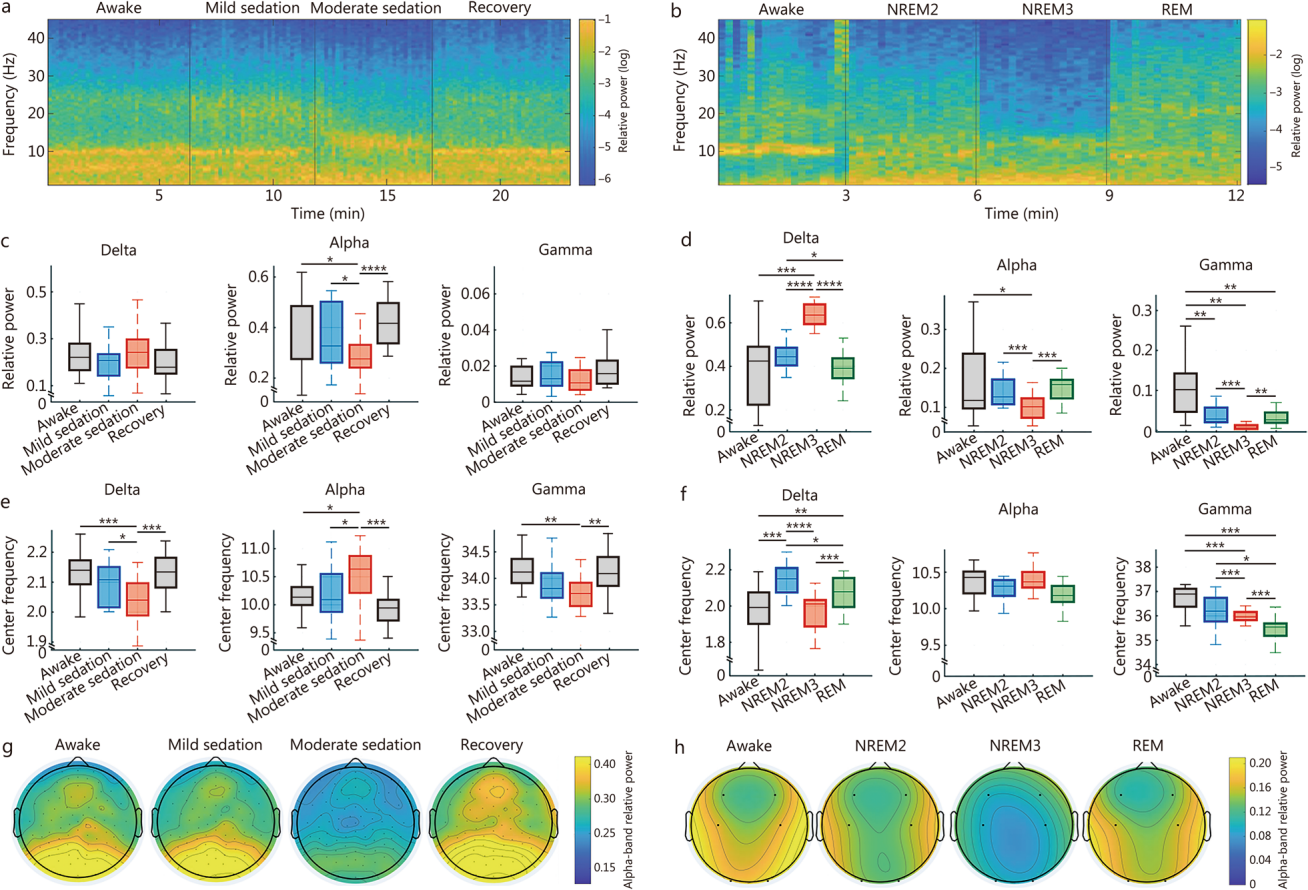
Periodic activity across propofol-induced anesthesia. **a** Time-frequency representation (TFR) of an example participant across propofol-induced states: awake, mild sedation, moderate sedation, and recovery, whole-brain averaged. **b** TFR of an example participant across sleep: awake, non-rapid eye movement (NREM)2/3, and rapid eye movement (REM), whole-brain averaged. Changes in spectral power across propofol-induced states (**c**) and sleep states (**d**) after averaged across the whole brain. Center line (50th), box edges (25th, 75th), and whiskers (10th, 90th) indicate percentiles. Changes in center frequency across propofol-induced states (**e**) and sleep states (**f**) after averaged across the whole brain. Topographic maps of group-averaged alpha-band relative power across propofol-induced states (**g**) and sleep stages (**h**). Paired *t*-tests with FDR correction were applied to compare group differences. ^*^P < 0.05, ^**^P < 0.01, ^***^P < 0.001, ^****^P < 0.0001. FDR false discovery rate

As illustrated in Fig. 3a and b, the channel-averaged time-frequency representations reveal progressive spectral changes as sedation deepened or sleep stages advanced. Under propofol-induced moderate sedation, where most participants retained responsiveness to auditory stimuli [147], delta power did not increase significantly (Fig. 3c). A similar pattern was observed during NREM2, where delta power also showed no marked elevation. However, delta power rose globally during NREM3, and returned to awake-comparable levels in REM sleep (Fig. 3d). The center frequency of the delta band decreased in moderate sedation when compared to mild sedation (Fig. 3e) and in NREM3 compared to NREM2 (Fig. 3f), a phenomenon potentially linked to the emergence of high-amplitude SWA, and these slowing effects reversed during recovery and REM sleep. These observations support the view that widespread delta power elevation may mark LOC, particularly the loss of internal awareness.

The alpha power was markedly suppressed during propofol-induced moderate sedation and NREM3 sleep (Fig. 3c, d), especially in occipital regions (Additional file 2: Fig. S1). A clear posterior-dominant alpha pattern was consistently observable during awake states, recovery, and mild sedation, but diminished under moderate sedation (Fig. 3g) and NREM3 (Fig. 3h). Although frontal alpha power did not increase (Additional file 2: Fig. S1), a relative topographic reorganization occurred, with frontal regions contributing more strongly than posterior sites to alpha activity. The absence of alpha anteriorization may be attributed to several factors, such as the selected frequency band (spindle frequencies may be faster), or spatial sampling limitations due to electrode coverage. Moreover, a notable acceleration in alpha frequency can be observed in Fig. 3e. This shift aligns with previously reported patterns of low-frequency slowing and increased APF during sleep and anesthesia [196], collectively reflecting a disruption of coupling between fast and slow neural oscillations [88]. However, these changes in alpha activity did not occur during REM sleep, suggesting they may specifically index loss of internal awareness.

Furthermore, the center frequency of gamma oscillations consistently decreased under both propofol-induced sedation (Fig. 3e) and NREM3 and REM sleep (Fig. 3f). Gamma power was significantly reduced during both NREM and REM sleep (Fig. 3d). However, it remained unchanged during propofol-induced sedation (Fig. 3c), consistent with the above hypothesis that preserved or elevated activity in certain subtypes of gamma oscillations does not suffice to maintain conscious experience.

### Aperiodic neural dynamics of DOC patients

**Fig. 4:**
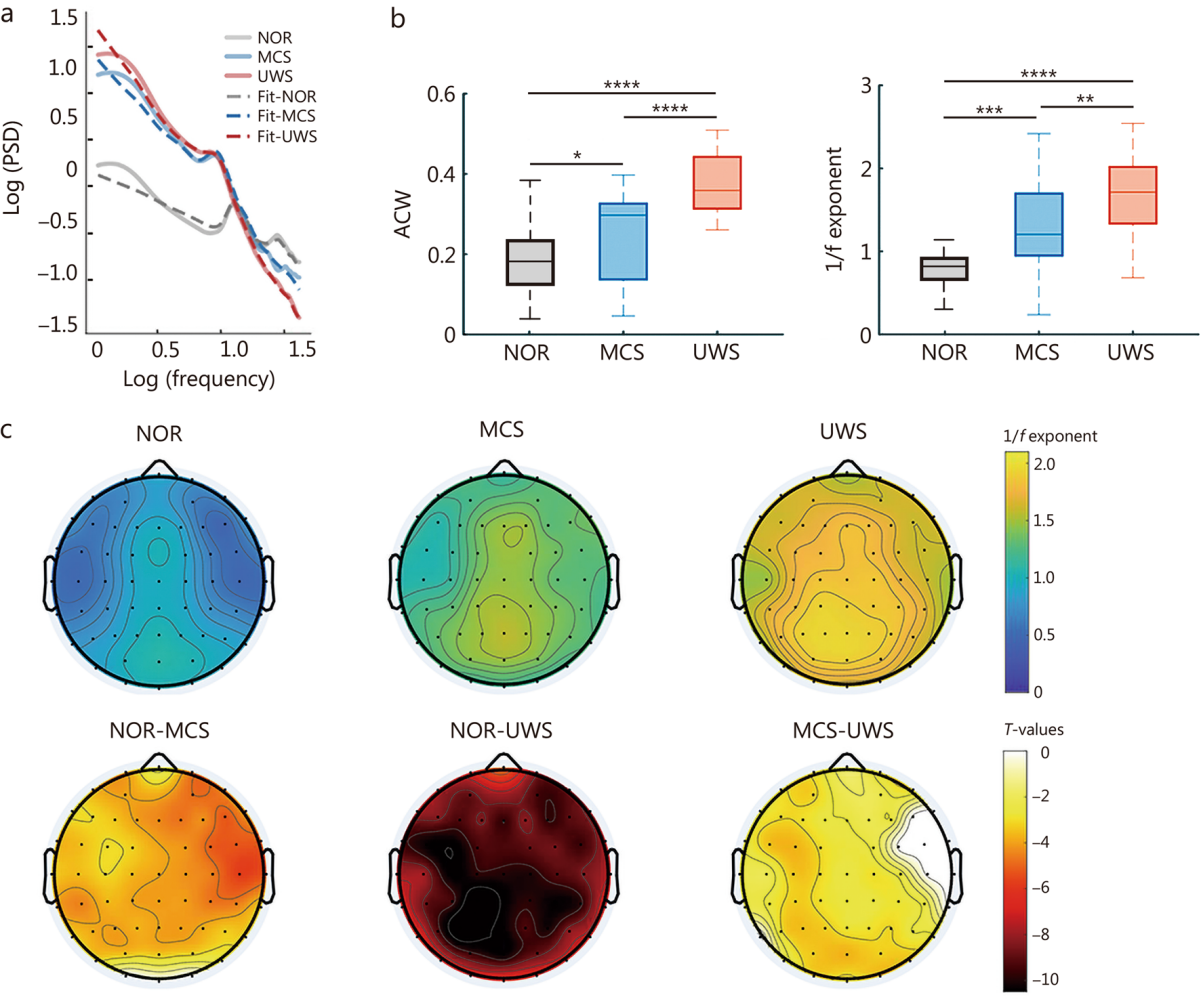
Aperiodic activity of patients with disorders of consciousness (DOC). **a** Power spectral density (PSD) and fitted 1/f structures between 1 and 30 Hz in normal control group (NOR), minimally conscious state (MCS), and unresponsive wakefulness syndrome (UWS). **b** Aperiodic components measured by the autocorrelation windows (ACW) and 1/f exponent. The values were averaged across all brain regions. Center line (50th), box edges (25th, 75th), and whiskers (10th, 90th) indicate percentiles. **c** Topographic maps of 1/f exponents and the corresponding *T*-values. Independent two-sample *t*-tests with FDR correction were applied between every 2 groups. ^*^P < 0.05, ^**^P < 0.01, ^***^P < 0.001, ^****^P < 0.0001. *T*-values are shown only for regions with significant between-group differences (*P* < 0.05, FDR-corrected). Non-significant regions are masked. FDR false discovery rate

Using dataset 3, neural aperiodic dynamics were evaluated in patients with DOC through time-domain (ACW) and frequency-domain (1/f exponent) metrics. ACW was calculated as the lag with which the autocorrelation function decays to 50% of its peak value. The 1/f exponent was measured by applying the FOOOF toolbox to the power spectrum in log-log space between 1 and 30 Hz (Fig. 4a). Both ACW and the 1/f exponent increased significantly as conscious level declined from the normal control group (NOR) to MCS and UWS (Fig. 4b). Notably, in NOR, the 1/f exponent displayed a posterior-to-anterior gradient, reflecting large-scale spatiotemporal hierarchy (Fig. 4c). However, this spatial gradient was disrupted in DOC patients, particularly in UWS, where the 1/f exponent topography became more homogeneous and less differentiated (Fig. 4c). As illustrated in Fig. 4c, patients with DOC exhibited a significant increase in the 1/f exponent across widespread cortical regions, compared with NOR. Within the DOC group, the difference between MCS and UWS was most pronounced in the parieto-occipital area, suggesting that this region may play a specific role in the fine-grained discrimination of consciousness levels.

### Connectivity and network organization

To examine FC and topological organization, dataset 3 was used to compute PCMI and to extract key graph metrics, including clustering coefficient, characteristic path length, and small-worldness.

**Fig. 5:**
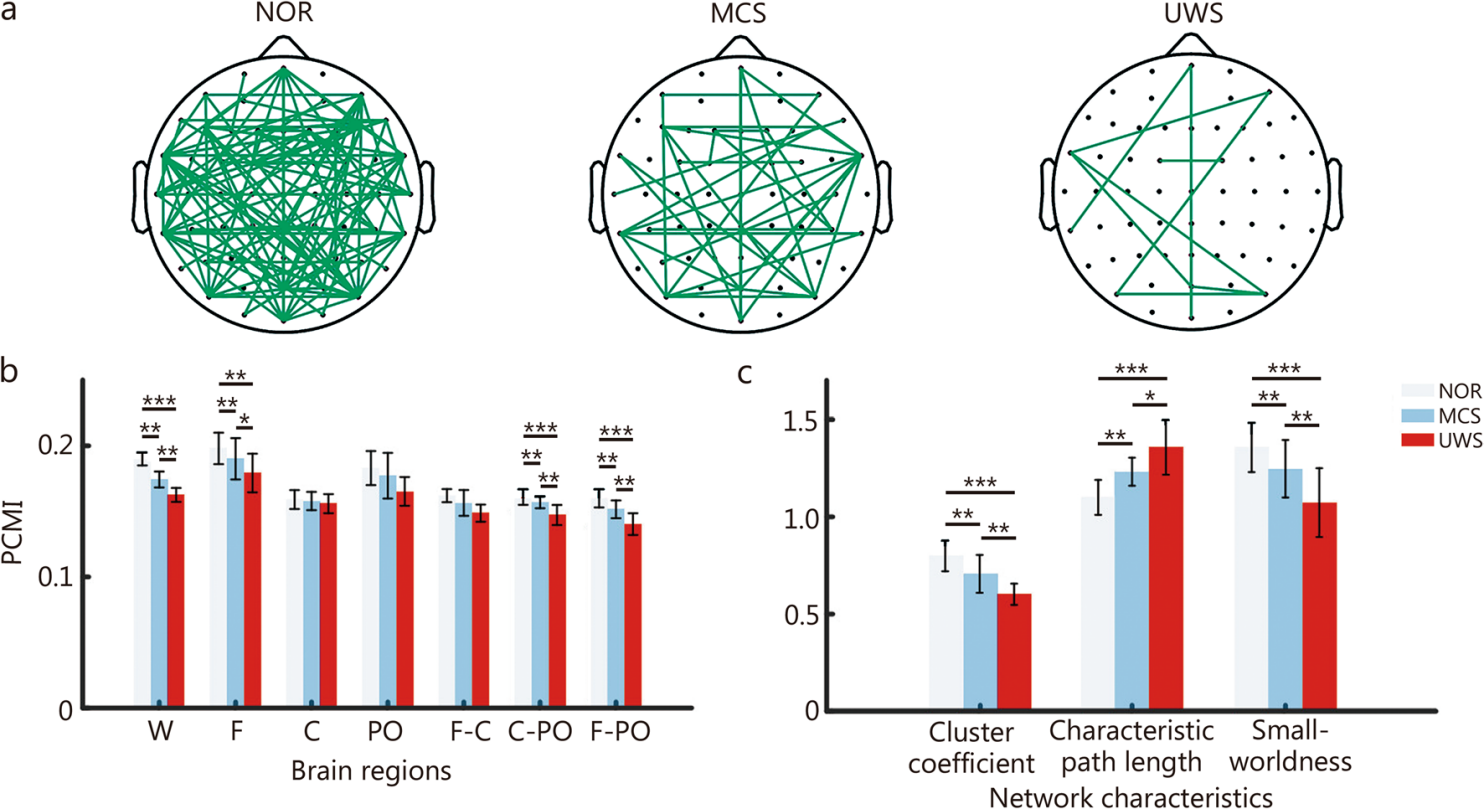
Functional connectivity and network organization. **a** Topographic map of the strongest 5% of permutation cross mutual information (PCMI) connections averaged in normal control group (NOR), minimally conscious state (MCS), and unresponsive wakefulness syndrome (UWS). **b** Regional averaged PCMI across whole brain (W), frontal (F), central (C), and parietal-occipital (PO) regions, as well as inter-regional PCMI between frontal and central (F-C), central and parietal-occipital (C-PO), and frontal and parietal-occipital (F-PO). **c** Network characteristics including clustering coefficient, characteristic path length, and small-worldness. Bars represent mean ± SEM. Independent two-sample *t*-tests with FDR correction were applied to compare group differences. ^*^P < 0.05, ^**^P < 0.01, ^***^P < 0.0001. SEM standard error of mean, FDR false discovery rate

As shown in Fig. 5a, healthy individuals exhibited significantly greater connectivity density than patients with MCS or UWS. Regional PCMI decreased gradually from NOR to UWS, particularly within frontal and frontoparietal regions (Fig. 5b). The global brain network in the NOR exhibited small-world topology, indicating an efficient balance between local specialization and global integration (Fig. 5c). In contrast, the small-worldness values decreased to approximately 1 in patients with UWS, suggesting a shift toward randomized network configuration as consciousness is impaired. This degradation in small-world organization was driven by a significant reduction in local clustering coefficient alongside a marked increase in characteristic path length (Fig. 5c). These findings indicate that consciousness may be associated with globally integrated and topologically efficient networks, while reduced consciousness is marked by disintegration and excessive local processing.

### Spatiotemporal microstate dynamics

**Fig. 6:**
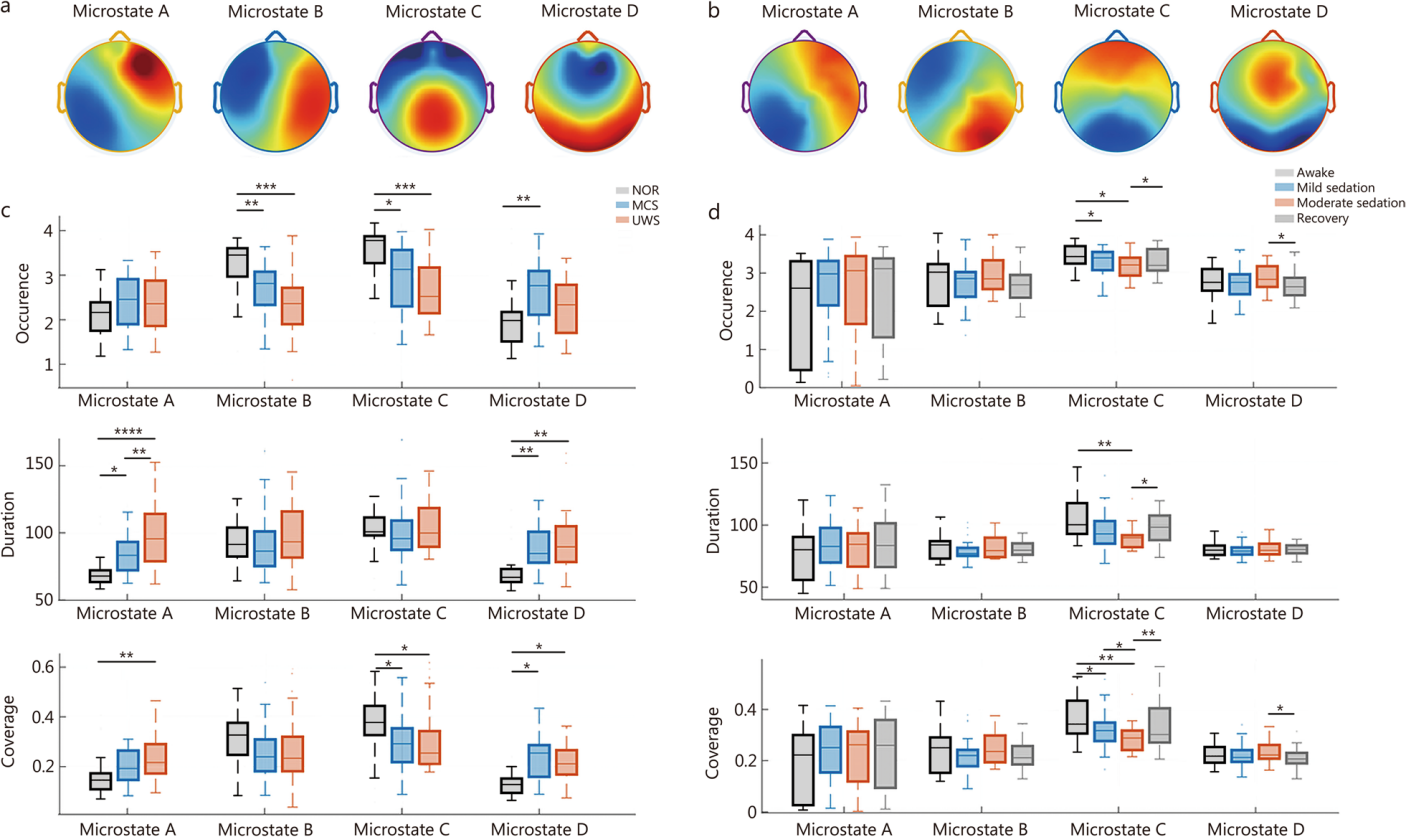
Altered spatiotemporal microstates in disorders of consciousness (DOC) and propofol-induced anesthesia. **a** Four microstates map (labeled as microstates A, B, C, D) in the normal control group (NOR), minimally conscious state (MCS), and unresponsive wakefulness syndrome (UWS). **b** Four microstates map (labeled as microstates A, B, C, D) across awake, mild sedation, moderate sedation, and recovery induced by propofol. **c** Box plots of occurrence rate, mean duration, and coverage for each microstate across NOR, MCS, and UWS. **d** Box plots of occurrence rate, mean duration, and coverage for each microstate across awake, mild sedation, moderate sedation, and recovery. Center line (50th), box edges (25th, 75th), and whiskers (10th, 90th) indicate percentiles. Independent two-sample *t*-tests were applied to compare group differences in DOC, and the Wilcoxon signed-rank test was applied for within-subject comparisons across propofol-induced states (FDR correction). FDR false discovery rate. ^*^P < 0.05, ^**^P < 0.01, ^***^P < 0.001, ^****^P < 0.0001

Microstate analysis offers a window into the spatiotemporal organization of global brain dynamics. Four microstate maps were identified at the group level [202] for dataset 1 and dataset 3 (Fig. 6). Based on the 4 standard categories described by Koenig et al. [203], these microstate maps were defined as microstate A, B, C, and D (Fig. 6a, b). Microstate A showed a left posterior-right anterior orientation, microstate B a right posterior-left anterior orientation, microstate C an anterior-posterior orientation, and microstate D showed a central maximum. Then, 3 parameters were calculated for each microstate, including occurrence (occurrence frequency of each microstate map), duration (the duration of each microstate map divided by the number of occurrences), and coverage (the duration of each microstate map divided by the total time).

Quantitative comparisons revealed distinct state-dependent alterations in microstate dynamics. As prior studies have reported [161, 163, 164], diminished levels of consciousness were generally associated with a reduced dynamic repertoire, prolonged microstate duration, and lower transition flexibility. Our results further discovered consciousness-specific effects in microstate dynamics (Fig. 6c, d).

Across both transitions toward moderate sedation or from NOR to DOC, microstate C exhibited a marked reduction in occurrence frequency and temporal coverage (Fig. 6c, d). Microstate C is associated with the salience network, encompassing regions such as the anterior cingulate cortex and bilateral inferior frontal gyri: areas critical for interoception and subjective representation [204]. Thus, microstate C activity may serve as a potential indicator for maintained conscious processing integrity, with its attenuation signifying impairment in higher-order cognitive functions, particularly executive control.

Conversely, microstate D showed the opposite pattern, with significant increases in both occurrence and coverage across both moderate sedation and DOC (Fig. 6c, d). Linked to a dorsal frontoparietal network that supports external attentional reorienting [204], enhanced microstate D activity may reflect a shift toward automated or compensatory network dynamics when top-down cognitive control, mediated by microstate C, is compromised. This antagonistic relationship underscores that consciousness is not a unitary “all-or-none” state but arises from dynamic interactions and balance among multiple functional networks. Consequently, consciousness assessment should focus on network equilibrium rather than relying on isolated metrics.

Condition-specific differentiations were particularly evident in microstate A activity (Fig. 6c, d). While UWS showed significantly prolonged microstate A duration compared to MCS, no significant changes in microstate A were observed under propofol-induced sedation. This divergence highlights the distinct network mechanisms underlying pharmacological vs. pathological consciousness impairment. On the other hand, microstate A is primarily associated with negative blood oxygen level-dependent activations in the bilateral superior and middle temporal gyri, regions implicated in phonological and auditory information processing [204]. During propofol-induced sedation, where behavioral responsiveness to auditory stimuli is preserved in partially retained awareness, microstate A parameters remain within normal ranges [147]. In contrast, abnormally prolonged microstate A duration likely reflects a more severe impairment of awareness in patients with UWS compared to those in MCS.

### Perturbation-response dynamics via TMS-EEG

TMS-evoked responses were analyzed using dataset 4. EEG responses to single-pulse TMS were decomposed into temporal principal components (PCs), capturing the underlying neural dynamics in a low-dimensional subspace (Additional file 2: Fig. S2).

**Fig. 7:**
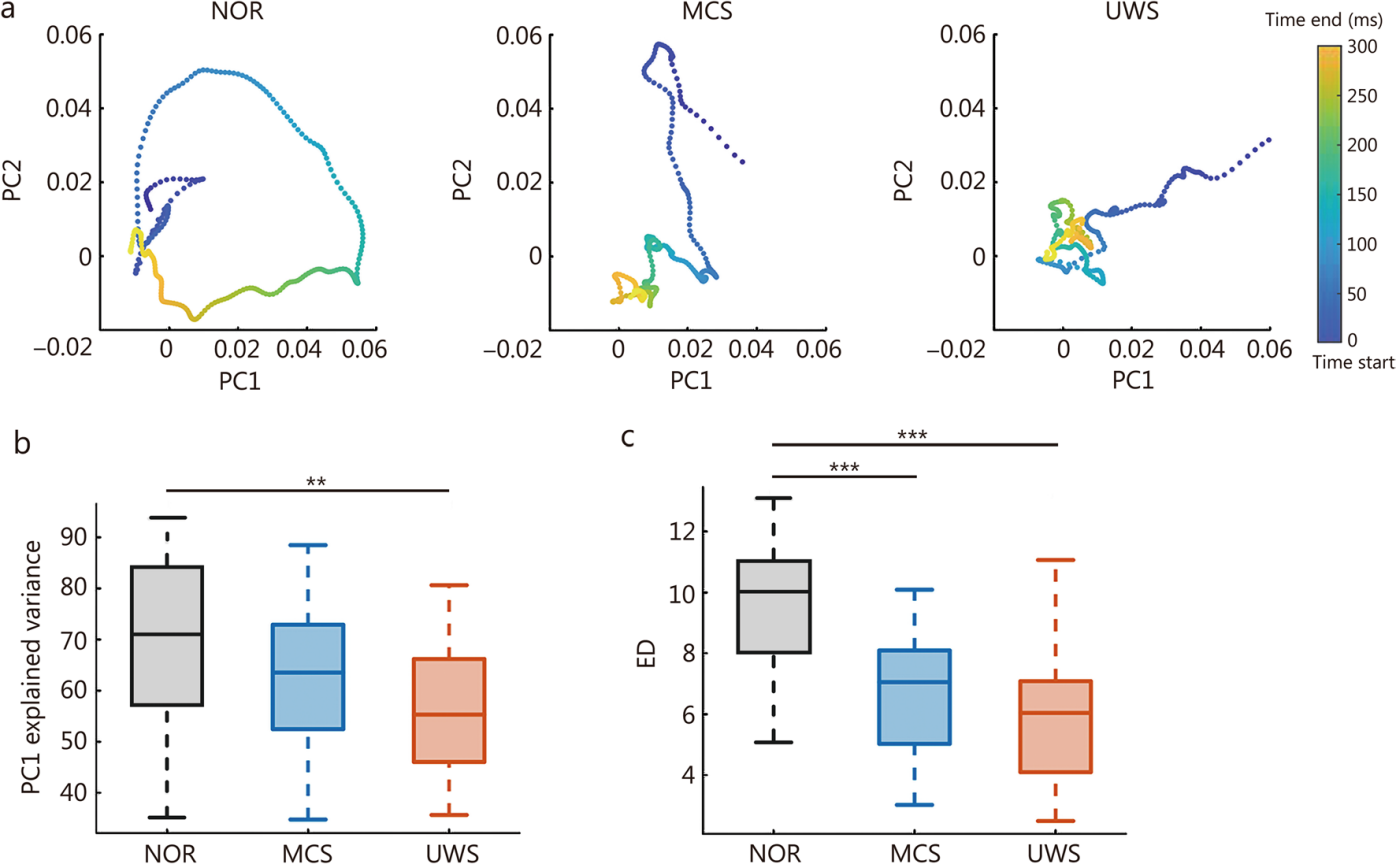
Perturbation-response dynamics of patients with disorders of consciousness (DOC). **a** Group-averaged trajectories of the transcranial magnetic stimulation (TMS)-evoked neural response (0–300 ms post-stimulation) projected into principal component (PC) space. Box plot of explained variance of PC1 (**b**) and effective dimensionality (ED) (**c**). Center line (50th), box edges (25th, 75th), and whiskers (10th, 90th) indicate percentiles. Wilcoxon rank-sum tests with FDR correction were applied to compare group differences. ^**^P < 0.01, ^***^P < 0.001. FDR false discovery rate, NOR normal control group, MCS minimally conscious state, UWS unresponsive wakefulness syndrome

Figure 7a shows the trajectory of neural activity in the PC1-PC2 space during the first 300 ms post-stimulation. In DOC, this trajectory was largely confined to the early response phase, suggesting a reduced capacity for sustained dynamic evolution compared to the normal control. ED was defined as the number of eigenvalues greater than 1 divided by the cumulative variance explained by eigenvalues larger than 1. Patients with DOC exhibited lower explained variance of PC1 (Fig. 7b) and lower ED (Fig. 7c). This progressive reduction in ED from NOR to DOC likely reflects a constrained repertoire of cortical spatiotemporal responses, pointing to diminished perturbational complexity as a neural signature of LOC.

## Discussion

### Distinguishing wakefulness, internal awareness, and external awareness

The lack of behavioral response does not necessarily correspond to LOC. When the relationship between consciousness and behavioral signs is inconsistent, locating objective indicators to calibrate brain conscious capacity is challenging. For instance, although a high PCI value can accurately identify residual consciousness in DOC patients, it also occurs during ketamine-induced anesthesia and REM sleep, even when behavioral responses are delayed. Internal awareness may be a key factor differentiating clinical conditions, highlighting the need to differentiate between “disconnected consciousness” and “LOC”.

We emphasize that states of unresponsiveness should be further separated into “disconnected consciousness” (REM sleep, ketamine-induced GA) and “LOC” (propofol-induced GA, deep NREM sleep, coma). By differentiating between these 2 states, several previously conflicting findings across different clinical contexts can be reconciled. Specifically, certain measures (e.g., delta/gamma activity, type I complexity, small-worldness) effectively detect complete LOC, while other measures (e.g., INT, type II complexity) may be related to general unresponsiveness. Meanwhile, measures such as the 1/f exponent and distance from criticality appear to track transitions into disconnected consciousness. Discriminating the neural substrates of connected consciousness, disconnected consciousness, and complete LOC is an intricate and subtle endeavor, posing a challenge that requires further conceptual and practical research.

It is crucial to note that the loss of the capability for external awareness may arise either from disrupted sensory connectivity (interruption of thalamocortical or corticocortical projection pathways in primary sensory areas) or from failed higher-order awareness processes involved in sensory information (disruption of top-down predictive and attentional pathways) [13]. However, constrained by methodological and technical limitations, distinguishing between these 2 mechanisms requires combining audio-visual perception paradigms. Future investigations may require more explicit operational demarcation of this distinction through multimodal neuroimaging protocols.

A significant challenge in calibrating internal awareness, particularly within the DOC population, stems from the inherent limitations of behavioral scales like the CRS-R. These tools rely predominantly on observable responses to external commands, rendering them inadequate for capturing subjective experiences such as dreaming, pain perception, or self-referential thought. To move beyond these limitations, future research should prioritize the multimodal integration of EEG-based sleep architecture in DOC, with particular emphasis on the properties and dynamics of dream sleep. Notably, dreaming occurs sporadically in NREM and is not guaranteed in REM sleep, preventing simple sleep-stage consciousness mapping. We here contrast dreamless NREM and dreaming REM as a simplified model, pending future dream-verified EEG studies. Subsequently, a validated neural correlate of internal awareness in communicative subjects (e.g., specific patterns of theta-gamma coupling or prefrontal high-frequency activity) could be established as a physiological benchmark to probe the capacity of internal awareness in non-communicative DOC patients. Ultimately, tracking these neurophysiological features in patients who eventually recover and can provide retrospective reports of their internal experiences would be crucial for confirming the predictive validity of such proposed biomarkers, thereby offering a more nuanced and profound insight into the spectrum of consciousness.

### Consciousness-specific effects: toward functional taxonomies

The subdivision of unresponsive states underscores the need to categorize “consciousness-specific” effects into those specific to wakefulness, internal awareness, and external awareness. A central implication of this tripartite framework is that not all neural features are equally diagnostic across all dimensions of consciousness. Neurophysiological signatures should be interpreted in one or several dimensions of consciousness (Table 2) [32, 33, 35, 36, 39, 47–50, 60, 65, 66, 88–91, 97–99, 102, 105–107, 114, 134, 137–141, 146, 152, 159, 185].

**Table 2:** Potential electrophysiological indicators corresponding to impairments in specific dimensions of consciousness in clinical conditions

Dimension of consciousness	Potential electrophysiological indicators	References
Wakefulness or External awareness	Collapse of the spatial gradients on natural frequency and intrinsic neural timescales	[88, 90]
	Reduced type II complexity	[105, 106]
	Asymmetry of frontoparietal forward and backward effective connectivity	[60, 138–141]
	Region-specific permutation cross-mutual information	[107]
	Delta phase synchrony	[98, 134]
	Frequency-specific small-world properties	[107, 146, 152]
Internal awareness	Delta dominance	[33, 35, 36, 39]
	Anteriorization of alpha power and coherence	[47–50]
	Reduced gamma power	[65, 66]
	Gamma hypersynchrony	[32, 137]
	Reduced Type I complexity	[91, 102]
	Reduced perturbational complexity index	[97, 99, 102]
General basis of consciousness	1/f exponent of power spectral density	[114]
	Functional geometry of the cortex	[159]
	Distance from criticality	[89, 106, 185]

For instance, the loss of feedforward connectivity and enhancement of feedback connectivity, alongside abnormal prolongation of INTs, were consistently observed during ketamine- and propofol-induced unresponsiveness [84, 87, 88], despite the wide variation in mechanisms of action of the anesthetics. Additionally, information capacity is reduced during all states of unresponsiveness across sleep, DOC, and anesthesia [105, 106], while randomness increases specifically during REM and ketamine-induced anesthesia [23, 107]. This suggests that internal awareness may preserve neural variability without supporting meaningful information capacity when brain states are at low wakefulness levels. Therefore, information capacity may reflect the level of wakefulness, and internal awareness specifically affects randomness. Meanwhile, certain features such as distance from criticality and the 1/f exponent may be jointly influenced by wakefulness level and internal awareness. In awake states, these features operate within a range associated with optimal brain function. During LOC or disconnected consciousness, they deviate in distinct directions [84, 96, 114], thereby helping to distinguish between connected, disconnected, and complete LOC. Furthermore, some features vary nonlinearly or ambiguously across consciousness levels depending on parameters, such as periodic activity, connectivity, cross-frequency coupling, and spatiotemporal patterns.

Given that conscious content generation is essentially dependent on fundamental consciousness levels [6], the three-dimensional consciousness framework constitutes a non-orthogonal architecture. This creates substantial challenges for separating dimension-specific effects, particularly in differentiating the neural mechanisms underlying diminished wakefulness vs. disrupted external connectedness, suggesting they may engage overlapping cortico-subcortical pathways. Studies combining auditory/tactile perception paradigms with DOC may help address the limitations of current guidelines by further dissociating wakefulness from external awareness.

The associations between EEG biomarkers and dimensions of consciousness proposed in this work are principally derived from the systematic integration of existing literature and theoretical inference. Although our clinical examples demonstrate the practical utility of various metrics in discriminating states of consciousness, they do not represent comprehensive empirical validation of the framework-biomarker relationships and, particularly do not include pharmacological models of disconnected consciousness (e.g., ketamine anesthesia). Future large-scale studies employing standardized datasets that encompass a broader spectrum of conscious states and multimodal paradigms, will be essential to rigorously evaluate the association strength and specificity of each biomarker to individual dimensions of consciousness, thereby establishing a clinically actionable reference.

### Aperiodic neural electrical activity associated with consciousness

The analysis of aperiodic neural electrical activity is a growing trend and should be considered in future studies. Currently, narrowband filtered periodic oscillations may contain aperiodic components, underscoring the need for more advanced signal separation tools. Moreover, while both periodic and aperiodic activities contribute to efficient information transmission and processing, their dynamic interactions and constraint relationships remain poorly understood. Higher-order characteristics, such as spatial topography and temporal dynamics of connectivity networks, achieve more consistent correlation with the level of consciousness. Future methodological development should therefore prioritize these high-order characteristics, covering broader spatial topology, more sustained temporal dynamics, and multiple networks.

### Complex neural dynamics for consciousness assessment

The diversity of electrophysiological signatures observed across altered states of consciousness may be unified within a systems-level theoretical framework centered on SOC. A growing body of evidence suggests that the conscious brain operates near the edge of order and disorder [170–172], where balanced stability-flexibility and integration-segmentation promote optimal information transmission, storage, and the formation of coordinated global dynamic patterns in the neural networks [108, 109, 160]. Conversely, during transitions into states such as complete LOC (e.g., deep NREM sleep, propofol-induced anesthesia), disconnected consciousness (e.g., REM sleep, ketamine anesthesia), or disorder of consciousness, the neural activity deviate from this optimal critical regime in distinct, state-specific directions.

However, the precise characterization of conscious criticality remains debated. Study based on power-law statistics proposed that the conscious brain may reside in a slightly subcritical regime, where neural avalanches approximate to a power-law distribution but with an exponential cutoff [205]. Therefore, key questions remain unresolved: 1) whether conscious brains operate precisely at criticality or in a near-critical regime; 2) how the two states of this critical boundary should be defined (e.g., order vs. disorder, integration vs. segregation, variability vs. stability); and 3) how to quantify the deviation from criticality, induced by physiological, pharmacological, or pathological perturbations.

While SOC provides a powerful interpretive lens, criticality analysis was not included in clinical validation datasets due to the complexity of its metrics and the diversity of data required to reach unified conclusions. The primary aim of this guideline is not to assert novel conclusions about consciousness and criticality, but to synthesize existing evidence into a coherent computational framework capable of guiding future research. Translating criticality metrics into clinically applicable tools for assessing consciousness will require large-scale, multi-modal datasets and collaborative validation efforts.

### Complementary modalities and multiscale integration

While EEG offers high temporal resolution and broad clinical applicability, it has limitations in source localization and subcortical access. Consciousness likely depends on interactions between cortical and subcortical hubs (e.g., thalamus, brainstem, basal forebrain), which may not be fully captured with surface EEG alone. For instance, a recent study reveals that higher-order thalamic nuclei in humans transiently gate conscious perception by driving the thalamo-prefrontal circuit [206]. Integrating EEG with other modalities, such as magnetoencephalography, intracranial EEG, or functional magnetic resonance imaging, can enhance spatial resolution and access to deeper structures. Multimodal approaches may also enable cross-validation of EEG-based metrics, helping to resolve discrepancies and improve mechanistic interpretability. Meanwhile, a critical limitation of the present discussion is its reliance on correlational evidence. To establish causal roles of EEG features, such as alpha anteriorization, in specific dimensions of consciousness, future studies should employ interventional approaches. Techniques such as electrical, magnetic, or ultrasonic stimulation of key brain regions could be used to selectively modulate these characteristic activities, while concomitant changes in behavioral and neural indices of consciousness are monitored.

Although our guideline outlines a comprehensive multi-dimensional EEG framework for consciousness assessment, its practical implementation faces several feasibility challenges. High-density EEG systems are ideal for spatial and network analyses, but are not always available in routine clinical care. Data collection requires careful artifact management and technical expertise, particularly in challenging populations like DOC patients. Furthermore, the computational burden of integrating advanced analyses (e.g., source localization, dynamic connectivity, perturbational complexity) may be prohibitive without specialized infrastructure. Future work should focus on developing streamlined, automated pipelines that prioritize the most robust biomarkers (e.g., PCI, alpha-delta power ratios) for feasibility in resource-limited settings.

## Conclusions

This study proposes an EEG-based methodological guideline for assessing consciousness by integrating multiscale biomarkers, including spectral dynamics, FC, spatiotemporal complexity, SOC, and TMS-evoked responses, across physiological, pharmacological, and pathological states. These measures collectively reveal a convergent neurophysiological signature of consciousness described by delta/gamma oscillations and peak frequency, a hierarchical organization of intrinsic timescales, metastable network interactions, balanced integration and segregation of spatiotemporal patterns, and proximity to criticality. These features are diminished or disrupted when one or more dimensions of consciousness are lost, supporting a unified neurophysiological architecture underlying diverse alterations of consciousness, and demonstrating their robustness and generalizability across diverse clinical contexts.

Translating this approach into clinical practice requires addressing key practical barriers, such as standardizing data acquisition, validating perturbational measures like TMS-EEG in heterogeneous patient groups, and defining normative ranges across ages and etiologies. Future work must prioritize large-scale, multimodal validation and open science initiatives to ensure reproducibility and diagnostic utility. This guideline provides a structured, empirically grounded foundation for the quantitative assessment of consciousness and neural correlates in clinical and research contexts.

## Supplementary Information

**Additional file 1**. Materials and methods.

**Additional file 2**. **Fig. S1** Topographic map of significant differences in alpha power. **Fig. S2** Principal components analysis of perturbation-response dynamics.

## Data Availability

Not applicable.

## Abbreviations

ACW: Autocorrelation window
AEC: Amplitude envelope correlation
APF: Alpha peak frequency
CRS-R: Coma recovery scale-revised
DCM: Dynamic causal modeling
DFA: Detrended fluctuation analysis
dFC: Dynamic functional connectivity
DOC: Disorders of consciousness
dPLI: Directed phase lag index
EEG: Electroencephalography
ED: Effective dimensionality
FC: Functional connectivity
FOOOF: Fitting oscillations and one-over-F
GA: General anesthesia
GABA: γ-Aminobutyric acid
GABA_A_: γ-Aminobutyric acid type A
GABA_B_: γ-Aminobutyric acid type B
GNWT: Global neuronal workspace theory
HADOs: High-amplitude delta oscillations
INT: Intrinsic neural timescales
LOC: Loss of consciousness
MCS: Minimally conscious state
NCC: Neural correlates of consciousness
NOR: Normal control group
NREM: Non-rapid eye movement
PAC: Phase-amplitude coupling
PCI: Perturbational complexity index
PCMI: Permutation cross-mutual information
PLI: Phase lag index
PLV: Phase locking value
PSD: Power spectral density
REM: Rapid eye movement
SOC: Self-organized criticality
STE: Symbolic transfer entropy
SWA: Slow-wave activity
TMS: Transcranial magnetic stimulation
UWS: Unresponsive wakefulness syndrome
wSMI: Weighted symbolic mutual information
