# Supplementary material for: A methodological guideline for consciousness assessment via neural electrophysiological activity
Source: Mil Med Res. 2025 Dec 12;12:90. doi: 10.1186/s40779-025-00682-4 (PMC12699880; doi:10.1186/s40779-025-00682-4)
Supplement: Supplementary file 1 — Additional file 1. Materials and methods. [file 40779_2025_682_MOESM1_ESM.pdf]

Materials and methods

Materials

Dataset 1. Electroencephalography (EEG) during propofol-induced sedation

Dataset 1 comprises EEG data collected from 20 healthy participants [11 females; age = 30.85 ± 10.98years] recorded during propofol-induced sedation [1]. Throughout the procedure, a computerized syringe driver (Alaris Asena PK, Carefusion, Berkshire, UK) was used to meticulously regulate the infusion of propofol and sustain target concentrations of 0.6 μg/ml for mild sedation and 1.2 μg/ml for moderate sedation. EEG was recorded using a 128-channel amplifier at a sampling rate of 250 Hz and with reference to the vertex electrode. All participants had their eyes closed during EEG data collection. At each stage of sedation, the participants were asked to perform a simple behavioral task: to quickly discriminate the type of auditory stimuli between buzz and noise. Trials were repeated 40 times, and the correct response rate and the average reaction time were then calculated. Blood samples (1 ml each) were taken at the beginning and end of the mild and moderate sedation states, as well as once at recovery to obtain the propofol levels in plasma. Conscious states were defined as awake, mild sedation, moderate sedation, and recovery according to drug dosage. Each state duration was approximately 7 min. Seven participants became behaviorally impaired at this simple task during moderate sedation, and 13 others remained responsive throughout, though their reaction times were impaired during sedation [1].

Dataset 2. EEG data during non-rapid eye movement (NREM) and rapid eye movement (REM)

EEG data were collected from 15 participants [10 females; age = 48.00 ± 17.01 years]. Each participant transitioned from wakefulness to sleep in a comfortable environment at night. EEG data were recorded by Compumedics Grael Polysomnography. The amplifier was connected to a 9-channel cap with

silver/silver chloride (Ag/AgCl) electrodes, and sampling of EEG data was conducted at 1024 Hz.

Dataset 3. Resting-state EEG data from patients with disorders of consciousness (DOC)

EEG data were collected from 90 participants, including 30 normal controls [15 females; age 48.36 ± 7.47 years] and 60 severely brain-injured patients with DOC. Within the DOC group, 30 patients were in the minimally conscious state [15 females; age = 49.67 ± 8.05 years] and 30 patients presented unresponsive wakefulness syndrome [15 females; age = 45.01 ± 9.08 years]. Ethical approval for this study was granted by the Ethical Committee of the Seventh Medical Center of the PLA General Hospital, China (2016-63). Written informed consent was obtained from NOR participants and the legal guardians of the DOC patients. Throughout the collection, a professional surgeon accompanied and observed the patients' state. Data collection was immediately suspended if the patient exhibited any of the following behavioral reactions: alterations in clinical status, increased muscle tension, or persistent teeth clenching.

EEG data were recorded by MR-compatible BrainAmp amplifiers (Brain Products, Germany). The amplifier was connected to a 64-channel cap with Ag/AgCl electrodes, and sampling of EEG data was conducted at 2500 Hz.

Dataset 4. TMS-EEG data from patients with DOC

TMS-EEG data were also collected from the cohort of 90 participants contributing to dataset 3. The F3 location, as specified in the international 10/20 system, was selected as the target position. The resting motor threshold (RMT) was measured in the motor cortex, and the stimulation intensity was set to 90% of the RMT. The RMT was measured within the motor cortex, and the stimulation intensity was subsequently calibrated to 90% of the RMT to effectively minimize electromyographic interference while eliciting a robust cortical response. The TMS-EEG session was carefully designed

to encompass 200 single-pulse TMS trials. The inter-trial intervals were randomized, ranging from 1.7 s to 2.3 s, to reduce potential habituation effects and enhance the signal-to-noise ratio. This rigorous trial configuration ensured the collection of high-quality TMS-EEG data, facilitating a comprehensive analysis of the cortical dynamics in response to the stimulation protocol.

EEG data were recorded by MR-compatible BrainAmp amplifiers (Brain Products, Germany). The amplifier was connected to a 64-channel cap with Ag/AgCl electrodes, and sampling of EEG data was conducted at 2500 Hz.

Methods

Data preprocessing

Dataset 1. Electrodes on the neck, cheeks, and forehead, which tended to contribute most of the movement-related noise, were excluded, and a total of 91 channels were involved in the following analysis. The channel locations are illustrated in **Additional file 1: Fig. S1**. EEG data then underwent a thorough preprocessing and analysis process. Four-second EEG epochs were first visually inspected to exclude those with artifacts like noise or eye movements. Following this, the data were resampled to 250 Hz and filtered with a 1 − 45 Hz bandpass, removing components related to eye movements and muscle activity via independent component analysis. Subsequently, the data were re-referenced to the average of all channels. The EEG data at each stage were segmented with a 10-second window and an overlap rate of 0 for in-depth analysis. This concise approach ensured the robustness and relevance of the results regarding the effects of propofol-induced sedation on brain activity and consciousness states.

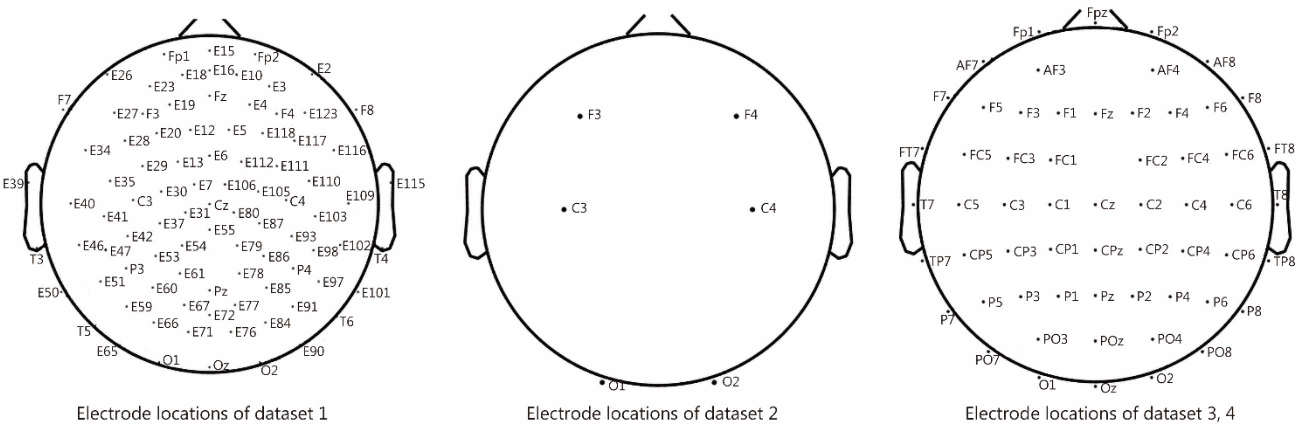

Fig. S1 Electrode locations of datasets 1 – 4. Dataset 1: high-density whole-scalp coverage. Dataset 2: sparse bilateral montage focused on frontal (F3, F4), central (C3, C4), and occipital (O1, O2) regions. Datasets 3, 4: standard-density array with comprehensive electrode nomenclature

Dataset 2. Excluding the ground and reference electrodes, a total of 6 channels were involved in the following analysis. The channel locations are illustrated in **Additional file 1: Fig. S1**. EEG data then underwent a thorough preprocessing and analysis process. Four-second EEG epochs were first visually inspected to exclude those with artifacts like noise or eye movements. Following this, the data were resampled to 250 Hz and filtered with a 1 − 45 Hz bandpass, removing components related to eye movements and muscle activity via independent component analysis. Subsequently, the data were re-referenced to the average of all channels. Each participant had at least 3 min of spontaneous EEG data recorded in the awake, NREM, and REM states. Consistent with dataset 1, the EEG data at each stage were segmented with a 10-second window and an overlap rate of 0 for in-depth analysis.

Dataset 3. Excluding the ground, reference, Lz, TP9, and TP10 electrodes, a total of 59 channels were involved in the following analysis. The channel locations are illustrated in **Additional file 1: Fig. S1**. The preprocessing pipeline consisted of 4 steps: (1) segmenting the data into 4-second epochs and visually rejecting the epochs contaminated by line noise, muscle activity, or eye movements; (2) resampling EEG data to 256 Hz and applying a 1 − 45 Hz bandpass filter; (3) performing independent

component analysis and removing components related to eye movements, blinks, or muscular activity; and (4) re-referencing data to the average of all channels. Three-minute continuous resting-state data were selected from each participant for further analysis. Note that in the microstate analysis, to be consistent with dataset 1, the sampling rate was changed to 250 Hz.

Dataset 4. Excluding the ground, reference, Lz, TP9, and TP10 electrodes, a total of 59 channels were involved in the following analysis. The channel locations are illustrated in **Additional file 1: Fig. S1**. The TMS-EEG data underwent a comprehensive preprocessing protocol. Initially, TMS pulses were precisely detected and marked, and corresponding epochs spanning 2000 ms (from -1000 to 1000 ms relative to the TMS pulse) were created to capture the neural activity surrounding the stimulation. These epochs were then subjected to rigorous visual inspection to exclude any containing noise, muscle activity, or eye movements, ensuring data quality. The data were subsequently resampled to 1000 Hz and filtered through a 1 – 80 Hz bandpass filter to isolate the pertinent frequency range. The TMS pulse artifact, which can distort the EEG signal, was carefully removed and replaced with cubic interpolation to maintain signal integrity. Independent component analysis was then conducted to identify and eliminate components related to eye movements, blinks, or muscular activity, further purifying the dataset. Finally, the EEG data were re-referenced to the average of all channels to provide a stable reference point. All analyses presented in this manuscript focused on the EEG data segment from 100 ms pre-stimulus to 330 ms post-stimulus, capturing both the early and late phases of cortical responses to the TMS stimulation, thereby facilitating a thorough examination of the elicited neural dynamics.

EEG data analyses

Periodic analysis. Power spectral density (PSD) was computed using the Fourier transform with a sliding window of 10 s without overlap. The relative power and center frequency were extracted across delta (1 − 4 Hz), alpha (8 − 13 Hz), and low gamma (30 − 45 Hz) bands.

Aperiodic analysis. Two key metrics of neural temporal structure were computed: the autocorrelation window (ACW) and the 1/f exponent, using non-overlapping 10-second EEG epochs. The ACW was defined as the time lag with which the autocorrelation function of the broadband EEG signal decays to 50% of its peak value. This measure captures the temporal persistence of neural activity at a global scale. The 1/f exponent was estimated using the FOOOF toolbox to parameterize the aperiodic component of the PSD. Each spectrum was fitted in log-log space across the 1–30 Hz frequency range, following the removal of oscillatory peaks. This exponent characterizes the balance between slow and fast fluctuations in the neural signal.

Connection and network property. Liang et al. [2] introduced genuine permutation cross-mutual information (PCMI), an improved mutual information algorithm that combines permutation entropy and mutual information algorithms to quantify functional connectivity between 2 time-series signals. The algorithm removes spurious correlations based on statistical differences between surrogate and original data, thereby more accurately reflecting the dependent information between 2 signals.

This study employed the PCMI method to characterize cortical connectivity changes. The computational framework for PCMI is detailed as follows:

1) Phase space reconstruction. EEG signals x_t and y_t were embedded into m-dimensional vectors using time-delay reconstruction with parameters $m = 3$ and delay $\tau = 1$:

$$X_t = [x_t, x_{t+\tau}, \dots, x_{t+(m-1)\tau}],\ Yt = [y_t, y_{t+\tau}, \dots, y_{t+(m-1)\tau}]$$

2) Ordinal pattern encoding. Vector components were sorted in ascending order to generate symbolic sequences S_{n} and S_{q}, yielding $m!$ possible patterns per vector.

3) Entropy computation. Marginal entropy was calculated from probability distributions:

$$H(X) = -\sum p(x) \log p(x),\ H(Y) = -\sum p(y) \log p(y)$$

4) Joint entropy calculation. Joint entropy derived from p(x, y):

$$H(X, Y) = -\sum p(x, y) \log p(x, y)$$

5) PCMI core formula.

$$\mathrm{PCMI}(X, Y) = H(X) + H(Y) - H(X, Y)$$

6) Surrogate data testing. Fifty surrogate pairs ($x_{t\mathrm{surr}}$, $y_{t\mathrm{surr}}$) were generated via Iterative Amplitude Adjusted Fourier Transform (IAAFT), with *c* computation of the orresponding $\mathrm{PCMI}_{\mathrm{surr}}$.

7) PCMI calculation based on statistical thresholding. Original vs. surrogate distributions were compared using Wilcoxon signed-rank test (one-tailed). If the null hypothesis is rejected ($H_0 = 1$ and $P < 0.001$), $\mathrm{PCMI} = \mathrm{PCMI}_{\mathrm{original}}$, otherwise: PCMI = 0.

8) Connectivity matrix analysis. Constructed 60 × 60 symmetric matrix, threshold = 70th largest PCMI value (connections below threshold were zeroed);

9) Calculation of different level PCMI. Global: mean PCMI of all connections. Intra-regional: mean within brain region; Inter-regional: mean between brain regions. The regional averaged connectivity in text was computed based on the following brain region parcellation: the frontal lobe (FP1, FP2, FZ, F3, F4, F7, F8, FC1, FC2, FC5, and FC6), central lobe (CZ, C3, C4, T3, T4, CP1, CP2, CP5, and CP6), and parietal-occipital lobe (PZ, P3, P4, P7, P8, PO3, PO4, PO7, PO8, Oz, O1, and O2).

To quantify the topological properties of the whole brain functional network, the following graph-theoretical metrics were computed. Characteristic path length is defined as the average of the shortest path lengths between all pairs of nodes in the network. This metric reflects the efficiency of global information integration. The clustering coefficient is measured for each node as the ratio of existing triangles formed with its neighbors to the maximum possible number of such triangles. The network's average clustering coefficient was then obtained by averaging across all nodes, indicating the level of local interconnectivity. Small-world propensity is quantified by comparing the network's average clustering coefficient and characteristic path length to those of an equivalent random network. This

scalar metric assesses the balance between global integration and local segregation.

Microstate analysis. EEG microstate analysis was performed following standardized procedures [3]. For all groups, global field power (GFP) peaks were first extracted to optimize the signal-to-noise ratio and were then clustered using a modified K-means clustering algorithm, which disregards topographic polarity, to derive a unified set of microstate templates applied consistently across the entire dataset [4]. The template derivation process involved iterative updating through the averaging of GFP maps showing the highest correlation with each template, while global explained variance (GEV) was computed to evaluate template quality. Iterations continued until GEV stability was achieved. The optimal number of microstate templates was defined as the minimal number of microstate maps that explain the maximal variance of a given EEG data set via the cross-validation criterion and GEV. These 4 group-level template maps were back-fitted to the individual EEG data with temporal smoothing (30 ms window) and a correlation threshold of 0.5 for segment labeling, thus generating continuous microstate sequences for subsequent analysis. From these labeled sequences, standard microstate metrics were computed including occurrence frequency per second, mean duration, and temporal coverage percentage for each microstate template.

Perturbation-response dynamics. The global dynamic range of TMS-evoked neural activity can be obtained by principal component analysis (**Additional file 2: Fig. S2**) of the time series from all channels ($N_{Channel} \times N_{Time}$). In the temporal dimension, eigenvalue decomposition was performed on the whole-brain neural activity evoked by TMS. We first focused on the first 2 principal components (PC1 and PC2). The eigenvectors of PC1 and PC2 characterize the trajectory of neural activity within a low-dimensional space (dynamics during 0–300 ms post-TMS are shown in **Fig. 7a**). The spatiotemporal dynamic range was then evaluated by calculating the effective dimensionality (ED), referring to the approach of Sheng et al. [5], defined as $ED = \frac{K}{V_K}$, where K is the number of principal

components with eigenvalue larger than 1, and V_K is the sum of explained variance of the first K largest principal components. An alternative is the maximum explained variance of the first principal component (PC1).

Statistical analysis

Paired *t*-tests were employed to compare group differences in the following electrophysiological measures: spectral power and peak frequency (**Fig. 3c, d, e, f**) and occurrence, duration, and coverage of microstates analyzed from dataset 1 (**Fig. 6d**). Two-sample *t*-tests were employed to compare group differences in the following electrophysiological measures: ACW and 1/f exponent (**Fig. 4b, c**), perturbational complexity index (PCMI) and network characteristics (**Fig. 5b, c**). Wilcoxon signed-rank tests were used for occurrence, duration, and coverage of microstates analyzed from dataset 3 (**Fig. 6c**). Wilcoxon rank-sum tests were used for explained variance and ED of principal components, with false discovery rate (FDR) correction for multiple independent comparisons (**Fig. 7b, c**). For topographic representations (**Fig. 4c** and **Additional file 2: Fig. S1**), statistical tests were performed on a per-electrode basis, followed by FDR correction across all channels. Only electrodes with corrected P-values < 0.05 are displayed with their corresponding T-values; non-significant regions are masked (T-value set to 0). Before all parametric tests, the Kolmogorov-Smirnov test was applied to assess the normality of the data distribution. Data pre-processing and analysis were performed using MATLAB (version 2020a; MathWorks, Natick, MA, USA) and the EEGLAB toolbox (version 13.4.4b).

References

1. Chennu S, O’Connor S, Adapa R, Menon DK, Bekinschtein TA. Brain connectivity dissociates responsiveness from drug exposure during propofol-induced transitions of consciousness. PLoS Comput Biol. 2016;12(1):e1004669.
2. Liang Z, Cheng L, Shao S, Jin X, Yu T, Sleigh JW, et al. Information integration and mesoscopic cortical connectivity during propofol anesthesia. Anesthesiology. 2020;132(3):504–24.
3. Michel CM, Koenig T. EEG microstates as a tool for studying the temporal dynamics of whole-brain neuronal networks: a review. NeuroImage. 2018;180:577–93.
4. Murphy M, Wang J, Jiang C, Wang LA, Kozhemiako N, Wang Y, et al. A potential source of bias in group-level EEG microstate analysis. Brain Topography. 2023;37(2):232–42.
5. Sheng J, Zhang L, Liu C, Liu J, Feng J, Zhou Y, et al. Higher-dimensional neural representations predict better episodic memory. Sci Adv. 2022;8(16):eabm3829.
