# Supplementary material for: A methodological guideline for consciousness assessment via neural electrophysiological activity
Source: Mil Med Res. 2025 Dec 12;12:90. doi: 10.1186/s40779-025-00682-4 (PMC12699880; doi:10.1186/s40779-025-00682-4)
Supplement: Supplementary file 2 — Additional file 2. Fig. S1 Topographic map of significant differences in alpha power. Fig. S2 Principal components analysis of perturbation-response dynamics. [file 40779_2025_682_MOESM2_ESM.pdf]

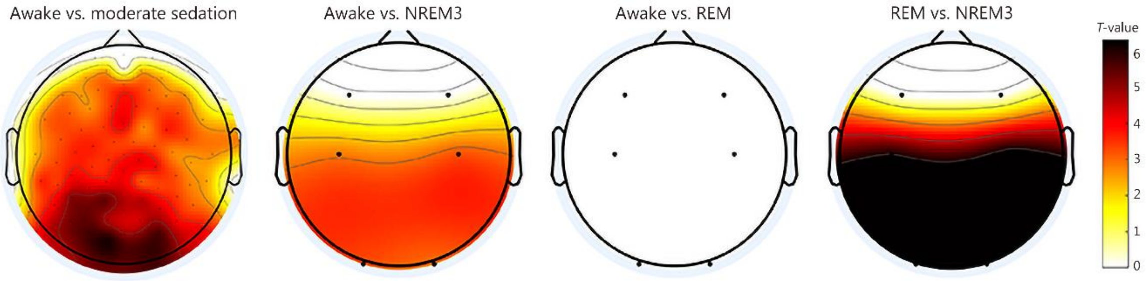

Fig. S1 Topographic map of significant differences in alpha power. Paired *t*-tests were employed to compare group differences between baseline and moderate sedation, awake and non-rapid eye movement 3 (NREM3), awake and rapid eye movement (REM), and REM and NREM3. *T*-values are shown only for regions with significant between-group differences ($P < 0.05$). Non-significant regions are masked

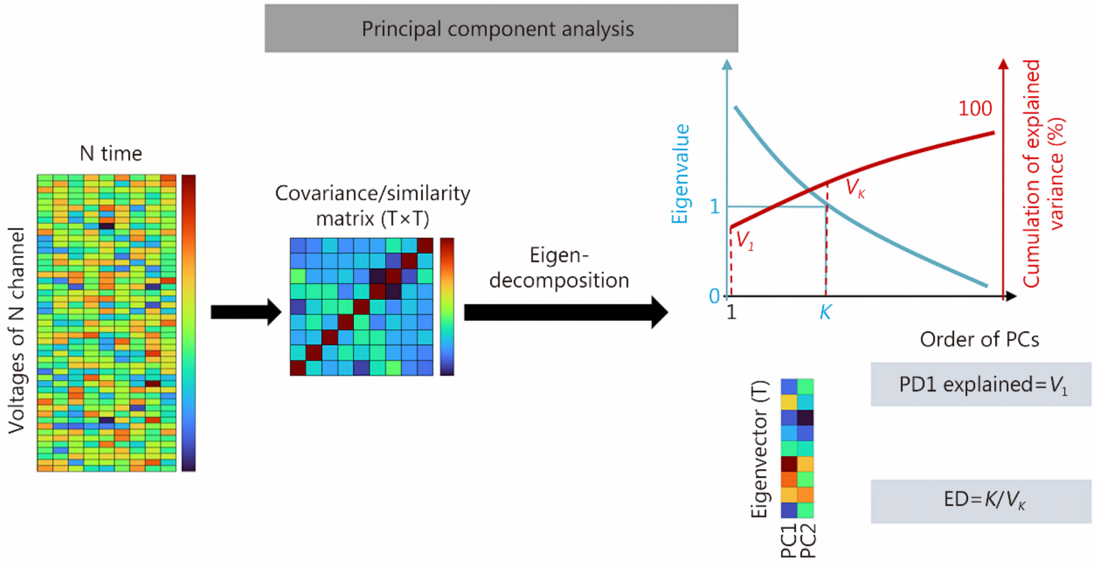

Fig. S2 Principal components analysis of perturbation-response dynamics. The process of calculating the explanatory variance of principal component (PC) and effective dimension (ED). K is the number of principal components with eigenvalue larger than 1, and V_K is the cumulation of explained variance of the first K largest principal components
